# Future direction of substrate‐based catheter ablation in Brugada syndrome and other inherited primary arrhythmia syndromes: Systematic review and meta‐analysis

**DOI:** 10.1002/joa3.12947

**Published:** 2023-11-01

**Authors:** Gusti Ngurah Prana Jagannatha, I Made Putra Swi Antara, Anastasya Maria Kosasih, Bryan Gervais de Liyis, Nikita Pratama Toding Labi, Wingga Chrisna Aji, Fanny Deantri, I Made Bagus Cahya Wibawa, Ida Bagus Satriya Wibawa, Jonathan Adrian

**Affiliations:** ^1^ Faculty of Medicine Udayana University/Prof. dr. I.G.N.G Ngoerah General Hospital Denpasar Bali Indonesia; ^2^ Division of Electrophysiology and Cardiac Pacing Department of Cardiology and Vascular Medicine, Faculty of Medicine Udayana University/Prof. dr. I.G.N.G Ngoerah General Hospital Denpasar Bali Indonesia; ^3^ Faculty of Medicine Sam Ratulangi University Manado North Sulawesi Indonesia; ^4^ Faculty of Medicine Muhammadiyah Yogyakarta University Yogyakarta Indonesia

**Keywords:** Brugada syndrome, catheter ablation, inherited primary arrhythmias syndromes, ventricular arrhythmia

## Abstract

**Background:**

Inherited Primary Arrhythmias Syndromes (IPAS), especially Brugada syndrome (BrS), have been associated with arrhythmogenic substrates that can be targeted through ablation. This meta‐analysis evaluated the outcomes of catheter ablation (CA) in different types of IPAS based on procedural guidance and location.

**Methods:**

A systematic search was conducted across multiple databases to identify studies reporting on ventricular arrhythmia (VA) events before and after CA in IPAS, including BrS, Long‐QT syndrome (LQTS), Early repolarization syndrome (ERS), and Idiopathic ventricular fibrillation (IVF). The primary outcomes were VA recurrence and VA burden, evaluated through conditional subgroup analysis. Procedural data were collected as secondary outcomes.

**Results:**

A total of 21 studies involving 584 IPAS patients who underwent CA were included. Following a mean follow‐up duration of 33.5 months, substrate‐based ablation demonstrated efficacy in reducing VA recurrence across all types of IPAS [RR 0.23; 95% CI (0.13–0.39); *p* < .001; *I* ^2^ = 74%]. However, activation guidance ablation was found to be effective only in IVF cases. Although recurrences still occurred, CA was successful in reducing VA burden [MD –4.70; 95% CI (−6.11–(−3.29); *p* < .001; *I* ^2^ = 74%]. The mean size of arrhythmogenic substrate was 15.70 cm^2^ [95% CI (12.34–19.99 cm^2^)], predominantly distributed in the epicardial right ventricular outflow tract (RVOT) in BrS cases and LQTS [Proportion 0.99; 95% CI (0.96–1.00) and Proportion 0.82; 95% CI ( 0.59–1.00), respectively].

**Conclusion:**

Substrate‐based CA has demonstrated effective prevention of VA and reduction in VA burden in IPAS cases.

## Graphical abstract

Ablation the specific sites of BrS and IPASs has been shown to diminish VA recurrence and VA burden. IPASs has been proven to possess arrhythmogenic substrates and activation and can be detected through a synergistic combination of substrate‐based and activation‐based mapping technique.

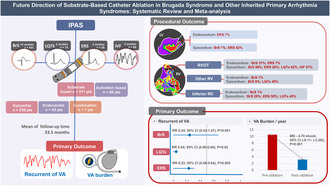

## 1 INTRODUCTION

Out‐of‐hospital cardiac arrest (OHCA) poses a significant medical, financial, and psychological burden, accounting for one‐third of mortality in adults under the age of 50.^1^ The ability to predict and ultimately prevent sudden cardiac death (SCD) has been described in the European Society of Cardiology Guidelines as the “philosopher's stone” of modern cardiology.^2^ Most SCD (90%) is caused by terminal arrhythmia events secondary to coronary artery disease, valvular heart disease, congenital heart disease, or infiltrative heart defects and cardiomyopathy.^3^ In contrast, the remaining 10% of SCD cases are associated with inherited primary arrhythmia syndromes (IPAS). These syndromes include Brugada syndrome (BrS), long‐QT syndrome (LQTS), catecholaminergic polymorphic ventricular tachycardia (CPVT), short‐QT syndrome (SQTS), early repolarization syndrome (ERS), and idiopathic ventricular fibrillation (IVF).^3^

The current treatment options for IPAS are primarily limited to implantable cardioverter‐defibrillator (ICD) implantation and pharmacological therapy. While ICD implantation is effective in terminating ventricular arrhythmias (VA) and preventing sudden death, it can lead to various psychological and physical challenges, as well as the potential discomfort of delivered shocks.^4^ Catheter ablation (CA) remains an option for the management of IPAS only when there is frequent recurrence of VA despite prior ICD implantation or failed pharmacological therapy. The effectiveness of CA in some IPAS cases still remains uncertain, and routine electrophysiology studies (EPS) have not been recommended in the current guidelines.^2^ However, recent studies have shed light on the presence of “arrhythmogenic substrates” in various IPAS, which are dispersed in both the epicardial and endocardial layers of the heart. These substrates have been observed even in asymptomatic patients who are suspected of triggering VA.^5^ Regrettably, the existing studies examining the effectiveness of CA in IPAS suffer from limitations. Additionally, the optimal approach for CA, whether it should be guided by substrate‐based or activation‐based methods, and the most suitable location for the procedure (epicardial and/or endocardial) remain subjects of ongoing debate in the scientific community. To address these uncertainties, the objective of this meta‐analysis was to comprehensively assess the impact of CA on VA recurrence and burden in IPAS population, taking into consideration to the particular procedures performed and the specific areas pinpointed for intervention.

## 2 METHODS

This systematic review adhered to the rigorous methodology outlined in the Preferred Reporting Items for Systematic Reviews and Meta‐analyses (PRISMA) guidelines, ensuring transparency and quality in reporting.^6^

### 2.1 Search strategy and selection criteria

A comprehensive search strategy was employed to identify relevant studies for inclusion in this systematic review. The following electronic databases were searched without language restrictions: MEDLINE (Medical Literature Analysis and Retrieval System Online) through PubMED, EMBASE (Excerpta Medical Database), and Cochrane Library. The search covered the period from the inception of these databases until March 31, 2023. The search strings used were: (Ablation) and ((Brugada) or (Early Repolarization) or (Idiopathic Ventricular Fibrillation) or (Catecholaminergic) or (Long‐QT) or (Short‐QT)).

All identified studies were screened by title and abstract. Four researchers independently identified studies that met the inclusion criteria (G.N.P.J., F.D., I.M.B.C.W., and I.B.S.W.). The inclusion criteria for this meta‐analysis were studies examining the outcome of catheter ablation in IPAS, including BrS, LQTS, CPVT, SQTS, ERS, and IVF, with the previously described diagnostic criteria.^2^ Upon thorough evaluation of the available literature, it was observed that no studies specifically addressing the outcomes of CA in relation to CPVT and SQTS were identified. Therefore, the discussion in this systematic review focused exclusively on the outcomes of CA in patients with BrS, LQTS, ERS, and IVF. Final eligibility was decided after the evaluation of full‐text publication. All disagreements are settled through discussion or involving a fifth referee (I.M.P.S.A).

### 2.2 Data extraction and quality assessment

Standard forms were used to extract the following information from each study: (i) study design and methodology; (ii) type of IPASs; (iii) population‐specific characteristics (patient inclusion criteria); (iv) mapping method (3D‐electroanatomical with or without activation mapping or conventional catheter activation mappings only); (v) ablation site (epicardial and/or endocardial); (vi) endpoint definition; (vii) baseline characteristics; and (viii) outcome as stated in the protocol of the current meta‐analysis.

For the systematic quality of included papers were evaluated using the recommended Newcastle‐Ottawa Scale (NOS) for observational studies.^7^ Investigations were classified as having low (<5 points), moderate (5–7 points), and high quality (>7 points).

### 2.3 Outcome measurement

The primary clinical outcomes in this study were the effectiveness of CA on VAs recurrency in IPASs by subgroup analysis based on IPAS types, VAs recurrency based on studies using the substrate‐based ablation method with subgroup analysis based on IPAS types, VAs recurrency based on studies using the activation‐mapping guidance only ablation method by subgroup analysis by type of IPASs, recurrency of VAs by subgroup analysis of ablation sites (Epicardial, endocardial, and combined epi‐ and endocardial), as well as VA burdens. The location of ablation was not separated by analysis with the IPASs subgroup due to limited data availability, as the majority of studies providing detailed information on ablation location focused on BrS. Substrate‐based ablation is defined as the ablation of an abnormal or low‐voltage area based on 3D‐electroanatomical mapping (3D‐EAM) with or without adjuvant activation‐mapping guidance. Activation‐mapping guidance ablation refers to a specific approach that targets the premature ventricular contractions (PVCs) responsible for ventricular fibrillation (VF). It involves eliminating endocardial abnormal potentials and/or ablating at the earliest activation sites determined through activation mapping, utilizing conventional or catheter mapping techniques without the use of adjuvant 3D‐EAM. All primary outcomes were extracted from pre‐ and post‐ablation data on the same subject. The secondary outcomes of this study included the proportion of early activation origins distributed by the type of IPASs, the proportion of arrhythmogenic substrate locations based on the type of IPASs, the mean percentage and area of low‐voltage areas, the proportion of SCN5A gene mutations, the proportion of ventricular arrhythmias inducibility during electrophysiology studies, and the proportion of procedural success rate. If a study included data and outcomes for patients who underwent anti‐arrhythmic drug (AAD) therapy without undergoing ablation, they were included in the secondary analysis to compare the recurrence of VAs between ablation and AADs.

VA recurrence in this study was defined as the occurrence of ventricular tachycardia (VT) or VF documented during the follow‐up period. Documentation of VA recurrence could include instances recorded on an ICD as appropriate shock or anti‐tachycardia pacing, 24‐h Holter monitoring, or 12‐lead electrocardiogram (ECG). VA burdens in this study were defined as the average number of VT or VF occurrences per year during the follow‐up period. These occurrences were recorded through various means, including the ICD as appropriate shock or anti‐tachycardia pacing, as well as on the 12‐lead ECG.

### 2.4 Data synthesis and analysis quality assessment

Data for a specific variable were included in the synthesis if it was reported in at least two of the included studies. Continuous variables were presented as the mean with a standard deviation (SD). Heterogeneity between the study populations was assessed using the *I* ^2^ statistic.^6^ Data across groups were summarized using the Mantel–Haenszel (M–H) risk ratio (RR) fixed‐effect model if *I* ^2^ < 25%. For *I* ^2^ values greater than 25%, the random‐effect model was employed.^8^ The subgroup analyses of the primary outcome were compared using the Z statistic. Funnel plots were used to evaluate publication bias as previously described.^8^ Analysis was carried out using Review Manager 5.4. and R Studio software.

## 3 RESULTS

### 3.1 Selection and description of studies

**FIGURE 1:**
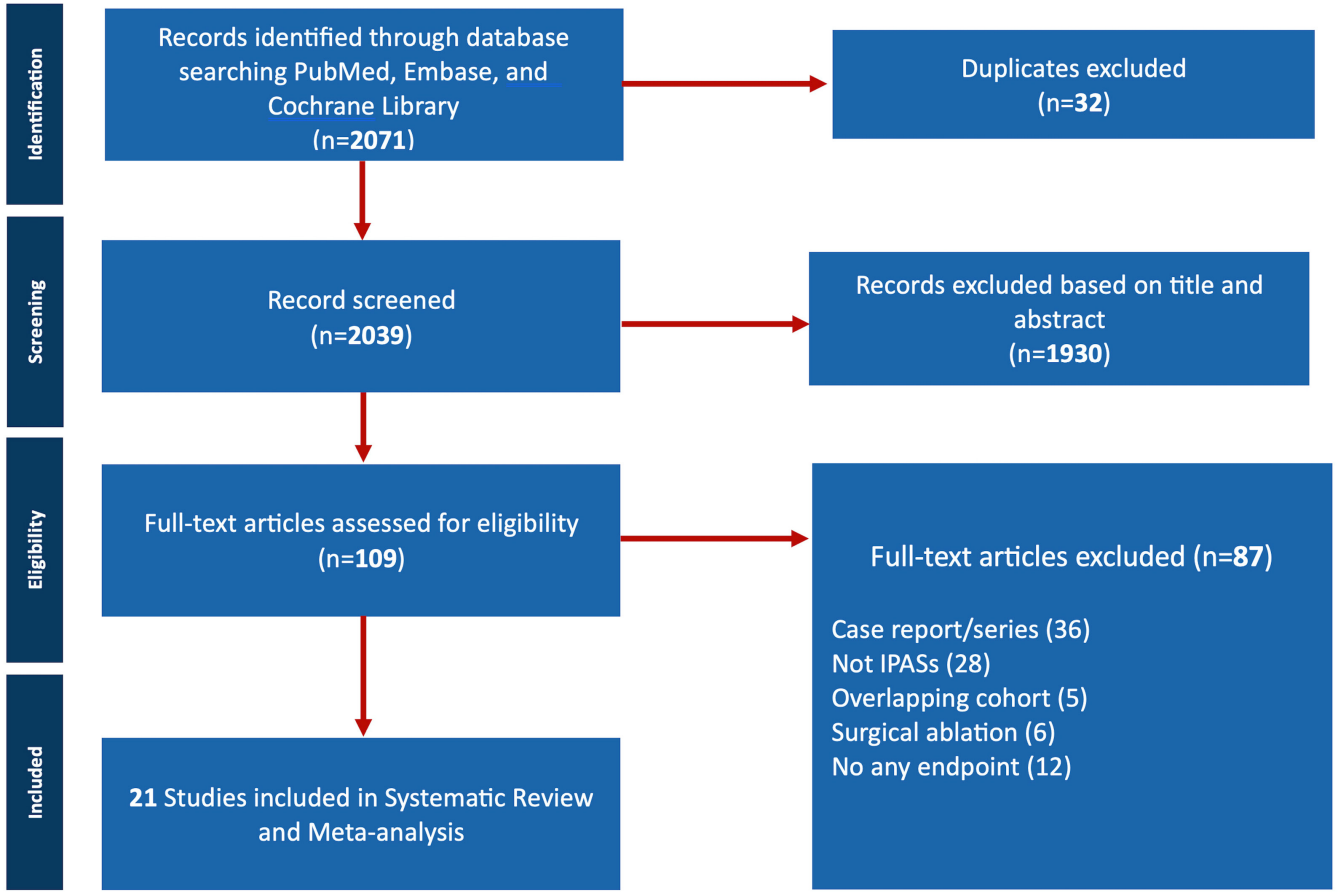
Preferred reporting items for systematic reviews and meta‐analyses (PRISMA) flow diagram. Depiction of selection of studies. IPASs, Inherited primary arrhythmia syndromes.

The study selection process is summarized in the PRISMA flow diagram presented in Figure 1. Initially, 2071 studies were identified through the primary search, and after removing duplicates, 2039 abstracts underwent independent screening by four investigators. A total of 87 studies were excluded from the analysis for multiple reasons. Inclusion of case series in our study has been undertaken to broaden the scope of our analysis, despite the inherent limitations associated with small sample sizes. A total of 21 studies were included in the data synthesis, as shown in Figure 1.

### 3.2 Characteristics of included studies

Tables 1 and 2 provide a comprehensive overview of the included studies, outlining their methodology, procedural characteristics, endpoints, and baseline data for study characteristics. It is important to note that the majority of the included studies primarily focused on BrS.^9^, ^10^, ^11^, ^13^, ^14^, ^16^, ^17^, ^18^, ^19^, ^21^, ^22^, ^23^, ^24^, ^26^ Procedurally, in determining the location of ablation on BrS, 11 studies used substrate‐based^9^, ^10^, ^13^, ^14^, ^16^, ^18^, ^19^, ^21^, ^22^, ^23^, ^26^and four studies used activation‐mapping guidance.^11^, ^17^, ^23^, ^24^ Whereas, in terms of the ablation approach, 10 studies in the BrS's population included outcome data for patients who received epicardial ablation,^9^, ^10^, ^13^, ^14^, ^16^, ^17^, ^18^, ^19^, ^21^, ^26^ seven studies included details of the outcomes of patients who received endocardial ablation^12^, ^14^, ^17^, ^21^, ^22^, ^23^, ^24^ and two studies had patient outcomes for patients who received combined endocardial and epicardial ablation in one procedure.^14^, ^21^ In the population with LQTS, each study utilized a substrate‐based and activation‐mapping guidance ablation method, as well as an epicardial and endocardial ablation approach, with equal representation across the included studies.^5^, ^12^ In the case of ERS, all included studies employed a substrate‐based ablation method, with the composition of epicardial and/or endocardial ablation being unknown. On the other hand, for IVF, all endocardial ablations were performed using the activation‐mapping guidance approach. In all included studies, radiofrequency energy was utilized for the ablation procedures.

**TABLE 1:** Summary of methodology and ablation details of included studies.

No	First author, year	Study design	Sample size and IPAS types				Description of procedures					
			Total population	Type of IPAS	Ablated	Not‐ablated	Inclusion criteria	Mapping	Substrates/VA provocation protocol	Ablation approach	Ablation energy	Definition of ablation site/substrate
1	Brugada, 2015^9^	Cohort Prospective, Single Center	14	BrS	14	0	BrS with symptoms attributable to VA or a high vulnerability for VA induction at electrophysiology study	3D‐EAM (CARTO 3)	VA induction was initially not performed, but substrate was identified under baseline and flecainide infusion (2 mg/kg in 10 min). After ablation, PVS (baseline and during flecainide infusion) from the RV apex, with 3 basal cycle lengths (600–500–400 ms), and ≤3 ventricular extra stimuli until refractoriness or to 200 ms, was used for VT/VF induction	Epicardial	RFCA	Low‐voltage areas (<1.5 mV)
2	Chung, 2017^10^	Cohort Retrospective, Single Center	15	BrS	15	0	BrS with history of aborted SCD or episodes of VT/VF	3D‐EAM	In the absence of spontaneous VA, rapid ventricular pacing and PVS up to three extra stimuli were performed with a catheter placed at the right ventricular apex and RVOT sequentially. If VA was still not inducible, intravenous isoprenaline 1–5 mg/min was infused to achieve at least 20% heart rate increment. If spontaneous VAs were not inducible during pharmacological provocation, the induction protocol was repeated	Epicardial	RFCA	Epicardial scar/low voltage zone
3	Haïssaguerre, 2002^11^	Cohort Prospective, Multi Center	27	IVF	27	0	IVF with high VA burdens or electrical storm on ICD	Conventional/Catheter Mapping	PVS with stimuli were twice the diastolic threshold and 2 ms in duration	Endocardial	RFCA	Earliest EGMs relative to the onset of the ectopic QRS complex. An initial sharp potential (10 ms in duration) preceding by 15 ms the larger and slower ventricular. electrogram during sinus rhythm represented a peripheral Purkinje component, whereas longer intervals indicated proximal Purkinje fascicle activation. Such a potential preceding ventricular activation during premature beats indicated that the latter originated from the Purkinje system, whereas its absence at the site of earliest activation indicated an origin from ventricular muscle
4	Haïssaguerre, 2003^12^	Cohort Prospective, Single Center	7	BrS	3	0	BrS/LQTs documented episodes of polymorphic ventricular tachycardia or VF	Conventional/Catheter Mapping	PVS using 1 to 3 programmed extra stimuli at the right ventricular apex and (if negative) from the RVOT at twice the diastolic threshold	Endocardial	RFCA	Earliest activation site of PVC
				LQTs	4	0			NA			
5	Haïssaguerre, 2022^13^	Cohort Retrospective, Multi Center	17	BrS	17	2	BrS presented sudden death due to documented VF	3D‐EAM (CARTO)	PVS from the RV apex and if unsuccessful from the LV using an initial basic CL of 600 ms and 400 ms drive trains, with up to three extra stimuli. In cases of non‐induction, burst pacing was performed at a CL of 250 ms decrementing by 10 ms steps to 160 ms or 2:1 capture	Epicardial	RFCA	Low‐voltage areas with low amplitude <1 mV (epicardial) or <1.5 mV (endocardial) and/or fractionation (3 components) of duration ≥70 ms
6	Kamakura, 2021^14^	Cohort Prospective, Single Center	16	BrS	16	0	BrS experienced at least 1 episode of VA	3D‐EAM (CARTO, Biosense‐Webster)	PVS was initiated from the RV apex followed by the LV by pacing with 2 basic cycle lengths (600 and 400 ms) with up to 3 extrastimuli until the refractory period was met	Endocardial and/or Epicardial	RFCA	Abnormal EGMs (signal bipolar amplitude <1.5 mV, signal duration 70 ms (onset to offset), signals with 3 components, split potentials, or late potentials)
7	Knecht, 2009^15^	Cohort Prospective, Multi Center	38	IVF	38	0	IVF experienced at least 1 significant event (either syncope, electrical storm, documented VF, or aborted sudden cardiac death) with documented PVC‐triggered VF on a 12‐lead ECG	3D‐ EAM (CARTO) and Conventional/Catheter Mapping	If required, PVC were induced by the use of pacing maneuvers (ventricular burst or extrastimuli) and/or isopro‐ terenol (1 to 4 g/kg/min) or adenosine (up to 40 mg) by intravenous infusion. If these measures were ineffective, pace mapping was used to identify the site of origin of clinical VPBs	Endocardial	RFCA	Earliest EGMs relative to the onset of the ectopic QRS complex during a ventricular ectopy. During sinus rhythm, the location of the Purkinje network was indicated by initial sharp potentials (10 ms in duration) preceding the QRS complex by 15 ms
8	Mamiya, 2021^16^	Cohort Retrospective, Single Center	27	BrS	11	16	BrS experienced episode of aborted sudden cardiac arrest, syncope, family history, or VF occurrence on an electrophysiological study and were implanted with an ICD	3D‐ EAM (CARTO)	PVS from the RV electrode catheter was used to evaluate VF inducibility. The pacing coupling interval was decreased to 180 ms with up to double extra	Epicardial	RFCA	Abnormal potential (local bipolar potentials with low voltage (≤1.0 mV); continuous and fractionated potentials with at least 2 distinct peaks; and long duration (≥150 ms) or distinct delayed potentials extending beyond the peak of the QRS complex on the surface ECG
9	Manero, 2015^17^	Cohort Retrospective, Multicenter	35	BrS	6	29	BrS with history of monomorphic VT	Conventional/Catheter Mapping and 3D‐ EAM (CARTO)	PVS with two to three drive cycles (600–500 and 400 ms, S1) and three extrastimuli (S2 to S4). The minimum coupling interval of premature beats was set to 200 ms	Endocardial and/or Epicardial	RFCA	Earliest activation site
10	Nademanee, 2019^18^	Cohort Retrospective, Multicenter	51	BrS	33	0	Symptomatic BrS/ERS either survived recurrent VF episodes or had cardiac or unknown syncope or agonal respiration during sleep	3D‐ EAM (CARTO) and Conventional/Catheter Mapping	PVS for VF induction (S1‐S1 at 600, 500, and 400 milliseconds and up to triple ventricular extrastimuli) was performed via the quadripolar catheter in the apex of the right ventricle (RV) or its outflow tract (RVOT)	Endocardial and/or Epicardial	RFCA	Abnormal electrogram (bipolar EGM that had low voltage (≤1 mV) and split EGM or fractionated EGM lasting ≥70 ms. or identifiable VF triggers
				ERS	18	0						
11	Nakamura, 2019^49^	Cohort Retrospective, Multi Center	13	IVF	13	0	IVF with recurrent episodes of sustained VF/Polymorphic VT	3D‐EAM (CARTO/CARTO3) and Conventional/Catheter Mapping	PVS with 1–4 extrastimuli of a minimum‐coupling interval of 180 ms, applied following 2 basic drives (600 ms and then 400 ms) and burst pacing from two ventricular sites was used to attempt to induce arrhythmias. Infusions or boluses of isoproterenol or epinephrine were used to attempt to induce PVCs in selected patients at the discretion of the operator	Epicardial	RFCA	PVCs that were observed or provokable and were consistent in morphology with the PVCs that had been suspected to initiate VF/Polymorphic VT based on previously recorded 12‐lead ECG
12	Noda, 2005^50^	Cohort Retrospective, Single Center	5	IVF	5	0	Spontaneous VF	Conventional/Catheter Mapping	If the target PVC was not recorded under baseline conditions, injection of isoproterenol, epinephrine, or methoxamine with or without PVS was used. Stimuli were twice the diastolic threshold and 2 ms in duration. Rapid burst pacing at multiple paced cycle lengths (pacing rate up to 250 beats/min) from right ventricular apex and the RVOT were performed in seven patients	Endocardial	RFCA	The site where the endocardial activation time during target VA was the earliest
13	Pappone, 2017^19^	Cohort Prospective, Single Center	135	BrS	135	0	Symptomatic BrS who had an ICD implanted	3D‐ EAM (CARTO 3)	NA	Epicardial	RFCA	Abnormal long‐duration bipolar electrograms. Defined as low‐frequency (up to 100 Hz) prolonged duration (>200 ms) bipolar signals with delayed activity extending beyond the end of the QRS complex
14	Pappone, 2023^5^	Cohort Prospective, Single Center	11	LQTs	11	0	LQTs frequently recurring spontaneous VF requiring ICD shocks	3D‐ EAM (CARTO 3)	VA induction was initially not performed. PVS from the RV apex was performed to assess ventricular arrhythmia inducibility after ablation, using 3 different cycle drives (600, 500 and 400 ms) up to 3 extrastimuli with a minimal coupling interval of 200 ms or until refractoriness	Epicardial	RFCA	Abnormal EGMs (fragmented and/or low‐voltage (<1.5 mV) signals exhibiting multiple components.)
15	Sadek, 2014^20^	Cohort Retrospective, Single Center	7	IVF	7	0	IVF patients underwent moderator band mapping, directly during the electrophysiologic study	3D‐EAM (CARTO/Ensite) and Conventional/Catheter Mapping	If spontaneous PVCs/VT were not identified at baseline, isoproterenol infusion (2–20 μg/min) and/or burst pacing from the RV apex, RV outflow tract, and/or high RA were performed in attempt to provoke VAs	Endocardial	RFCA	Sites with the earliest activation preceding the onset of the PVCs
16	Shelke, 2017^21^	Cohort Prospective, Single Center	5	BrS	5	0	BrS with recurrent ICD shocks for VT/VF	3D‐EAM (CARTO3 /Ensite NavX)	PVS with up to three extrastimuli were delivered from the RV apex and the RVOT using short‐long‐short sequence protocols up to ventricular effective refractory. When these protocols did not induce VT/VF, a protocol of burst pacing up to 300 ms from the RV apex and the RVOT was used. In case of non‐inducibility of VT/VF, these protocols were then repeated from the LV. In case of non‐inducibility of sustained VA from the RV and LV at baseline, induction was attempted during an infusion of Phenylephrine (0.1–0.5 mg bolus over 1 min followed by an infusion of 0.5–1 mg/kg/min	Endocardial and/or Epicardial	RFCA	Abnormal EGMs defined as fractionated potentials (continuous multi component EGM with amplitude <1 mV and duration >50 msec), split EGMs (two discrete EGMs separated by an isoelectric interval of >50 msec), isolated late potentials (EGMs inscribed entirely after the QRS complex) and low voltage EGMs (bipolar voltage <1.5 mv, filtered at 30–300 Hz with a notch filter at 50 Hz) in sinus rhythm
17	Sunsaneewiyatakul, 2012^22^	Cohort Retrospective, Single Center	10	BrS	4	6	BrS with frequent VF shocks and VF storms	3D‐EAM (EnSite)	PVS was performed at the RV apex and RVOT with 2 basic cycle lengths (600 and 400 milliseconds) and up to 2 ventricular extra‐stimuli (S2 down to an effective refractory period and S3 down to a minimum of 180 milliseconds)	Endocardial	RFCA	Region that had electrical activity occurring during J point to 60 (J + 60) milliseconds interval of the V1 or V2 of surface ECG was considered as the late activation zone
18	Talib, 2023^23^	Cohort Retrospective, Multicenter	123	BrS	21	102	BrS with recurrent ICD discharges or electrical storm resistant to medical therapy	3D‐EAM (CARTO/Ensite)	PVC provocation using sodium channel blocker with intravenous pilsicainide infusion (0.25 mg/kg for 5 min) was performed	Endocardial	RFCA	The earliest local electrogram relative to the onset of the QRS complex during a ventricular ectopy and abnormal electrogram (fractionated electrograms multicomponent with an amplitude of 50 ms), or isolated late potentials (inscribed entirely after the QRS complex), and low‐voltage electrograms (<1 mV)
19	Tokioka, 2019^24^	Cohort Prospective, Single Center	7	BrS	7	0	BrS without structural heart diseases ruled out by using echocardiogram, coronary angiogram, cardiac magnetic resonance imaging, and cardiac biopsy	3D‐EAM (CARTO/Ensite) and Conventional/Catheter Mapping	PVS with two drive cycles (600 and 400 ms, S1) and up to three extra stimuli (S2–S4) from the RV apex (RVA) and RVOT during continuous intravenous administration of propofol. The minimum coupling interval of premature beats was 180 ms in two extra stimuli and 200 ms in three extra stimuli. Overdrive pacing and administration of isoproterenol were attempted when VT was not induced	Endocardial	RFCA	Low Voltage area <1.5 mV on the bipolar voltage map or <3.8 mV on the unipolar voltage map
20	Voskoboinik, 2019^25^	Case series	10	ERS	3	7	History of syncope or VA	3D‐EAM and/or Conventional/Catheter Mapping	PVS	Endocardial and/or Epicardial	RFCA	Low voltage region
21	Zhang, 2016^26^	Cohort Prospective, Multi Center	11	BrS	11	0	BrS who presented with documented VT/VF and/or syncope	3D‐EAM (CARTO 3)	VT/VF induction was performed with decremental burst pacing or PVS with 3 basal cycle lengths (600–500–400 ms), and up to 3 ventricular extrastimuli until refractoriness or to 200 ms	Epicardial	RFCA	Abnormal electrograms region (prolonged‐duration electrograms and delayed conduction)

Abbreviations: BrS, Brugada syndrome, ECG, electrocardiography, EGM, electrogram, ERS, early repolarization syndrome, ICD, implantable cardioverter defibrillator, IPAS, inherited primary arrhythmia syndrome, IVF, idiopathic ventricular fibrillation, LV, left ventricle, LQTS, long‐QT syndrome, PVC, premature ventricular contraction, PVS, programmed ventricular stimulation, RFCA, radio frequency catheter ablation, RV, right ventricle, RVOT, right ventricular outflow tract, SCD, sudden cardiac death, VA, ventricular arrhythmia, VF, ventricular fibrillation, VT, ventricular tachycardia, 3D‐EAM, 3D electro anatomical mapping.

**TABLE 2:** Characteristics of population and definition of outcome.

No	First author, year	Type of IPASs	Age	Male (%)	Tested gene mutations (*n*)	AADs after ablation before VA recurrent (*n*, type)	Complication (*n*)	Death	Mean follow‐up time (month)	Definition of VAs recurrency
1	Brugada, 2015^9^	BrS	37.3	100	SCN5A (4)	NA	1 (Transient pericarditis)	0	4.5	Documented on ICD
2	Chung, 2017^10^	BrS	41.3	100	SCN5A (3)	NA	0	0	18.2	Documented on ICD
3	Haïssaguerre, 2002^11^	IVF	40.62	51.8	NA	0	NA	0	24	Documented on ICD
4	Haïssaguerre, 2003^12^	BrS	39.7	67	SCN5A (1)	0	NA	0	7	Documented on 24‐h Holter and/or 12‐lead ECG
		LQTs	37	50	KCNQ1, SCN5A, and HERG	1 (Beta‐blockers)			17	
5	Haïssaguerre, 2022^13^	BrS	45	100	NA	NA	NA	0	56	Documented on ICD and/or 12‐lead ECG
6	Kamakura, 2021^14^	BrS	44.5	93	SCN5A (9)	0	0	NA	25.1	Documented on ICD
7	Knecht, 2009^15^	IVF	42	55	NA	0	NA	0	61	Documented on ICD
8	Mamiya, 2021^16^	BrS	45.2	100	SCN5A (1)	0	0	NA	42	Documented on ICD
9	Manero, 2015^17^	BrS	43.4	77.1	SCN5A (28)	0	NA	0	69.4	Documented on ICD and/or 12‐lead ECG
10	Nademanee, 2019^18^	BrS	37.4	92.1	SCN5A (4)	0	1 (Hemopericardium)	1	31	Documented on Holter recording at 24 h
		ERS			SCN5A (0)					
11	Nakamura, 2019^49^	IVF	50	54	NA	NA	1 (Cardiac tamponade)	0	20.2	Documented on ICD and/or 12‐lead ECG
12	Noda, 2005^50^	IVF	42.4	NA	NA	1 (Beta‐blocker)	NA	0	54	Documented on ICD and/or 12‐lead ECG
13	Pappone, 2017^19^	BrS	39	71.1	SCN5A (32)	NA	5 (Epicardial effusion)	0	10	Documented on ICD and/or 12‐lead ECG
14	Pappone, 2023^5^	LQTs	44	54	KCNH2 (2), KCNQ1 (4), KCNE1 (1), and SCN5A (1)	11 (Beta‐blocker)	1 (Pericarditis)	0	12	Documented on ICD and/or 12‐lead ECG
15	Sadek, 2014^21^	IVF	42.1	85.7	NA	0	0	0	21.4	Documented on ICD and/or 12‐lead ECG
16	Shelke, 2017^21^	BrS	29	80	NA	NA	2 (Pericarditis and pericardial effusion)	1	47.8	Documented on ICD and/or 12‐lead ECG
17	Sunsaneewiyatakul, 2012^22^	BrS	25	100	NA	0	1 (RBBB during ablation)	0	25.5	Documented on ICD
18	Talib, 2023^23^	BrS	43	90.4	SCN5A (3)	0	1 (Steampop) and 2 (transient RBBB)	NA	56.14	Documented on ICD
19	Tokioka, 2019^24^	BrS	50	100	NA	NA	NA	NA	97.8	Documented on ICD
20	Voskoboinik, 2019^25^	ERS	30	80	NA	0	NA	0	53.76	Documented on ICD and/or 12‐lead ECG
21	Zhang, 2016^26^	BrS	47.6	100	SCN5A (2)	0	2 (self‐limited pericarditis)	1	25	Documented on ICD and/or 12‐lead ECG

Abbreviations: BrS, Brugada syndrome; ECG, electrocardiography; ERS, early repolarization syndrome; ICD, implantable cardioverter defibrillator; IPAS, inherited primary arrhythmia syndrome; IVF, idiopathic ventricular fibrillation; LQTS, long‐QT syndrome; RBBB, right bundle branch block.

The protocol for inducing VA after ablation was consistently described in all of the studies reviewed. This protocol involved using an EPS protocol and specific drugs such as flecainide, isoprenaline, or pilsicainide. However, it is noteworthy that two studies, specifically the one conducted by Pappone et al.^19^ in the BrS group and the study by Haïssaguere et al.^12^ in the LQTs group, did not provide details regarding the specific VA provocation protocol that employed (Table 1). The recurrence of VA was defined as the occurrence of VT or VF during the follow‐up period after ablation. However, in three studies, the use of ICD was not universal across all populations.

### 3.3 Risk of bias

The included studies demonstrated moderate to good quality overall, as indicated by their Newcastle‐Ottawa Scale (NOS) scores ranging from 7 to 8 (Table S1) and *I* ^2^ statistic for heterogeneity assessment between the study populations were available in Figure S2. However, it is important to note that none of the studies controlled for potential confounding variables that could influence the risk of VA. In terms of comparability, all studies are considered equivalent as they involve comparing events before and after ablation within the same subjects.

### 3.4 Data synthesis

A total of 21 studies, encompassing 584 patients, were included in the analysis. Among the population with IPASs, BrS constituted the majority, accounting for 77.2% of the overall IPASs population. The IPASs population predominantly consisted of males (81.1%), with an average age of 38.9 years (Table 2). In both the entire IPASs population and the BrS population specifically, approximately 25% of patients were confirmed to have the SCN5A gene mutation, [proportion 0.25; 95% CI (0.17–0.32); *I* ^2^ = 40%] and [proportion 0.25; 95% CI (0.19–0.30); *I* ^2^ = 17%], respectively (Figure S3).

### 3.5 Procedural outcomes

In patients who underwent 3D‐EAM, it was found that the distribution of arrhythmogenic substrates slightly varied between the types of IPASs (Figure S1). In the BrS group, the majority of arrhythmogenic substrates were found to be distributed in the epicardial right ventricular outflow tract (RVOT) and/or anterior right ventricle (RV), with low heterogeneity [proportion 0.99; 95% CI (0.96–1.00); *I* ^2^ = 28%]. Similarly, in LQTS, the dominant location with the highest distribution of arrhythmogenic substrates was the epicardial RVOT and/or anterior RV, accounting for 82% [proportion 0.82; 95% CI (0.59–1.00)]. Other arrhythmogenic substrate locations in LQTS included the inferior epicardial RV and other regions of the RV, each with equal proportions of 45% [proportion 0.45; 95% CI (0.16–0.75)].

Heterogeneity in the distribution of arrhythmogenic substrates in LQTS could not be estimated due to limited reporting in only one out of two studies utilizing 3D‐EAM. Meanwhile, in ERS, the distribution of arrhythmogenic substrates showed significant variation, not only limited to the RV [RVOT/anterior RV: proportion 0.26; 95% CI (0.00–0.82); *I* ^2^ = 88% and inferior RV: proportion 0.50; 95% CI (0.00–1.00); *I* ^2^ = 99%], but also extending to the left ventricle (LV) [proportion 0.42; 95% CI (0.06–0.77); *I* ^2^ = 29%]. In IVF, no arrhythmogenic substrate was found on either the epicardium or endocardium. The average size of the arrhythmogenic substrate was 15.70 cm^2^ [95% CI (12.34–19.99 cm^2^); *I* ^2^ = 89%], accounting for an average of 6.23% of the total heart surface area [95% CI (0.78–49.7%); *I* ^2^ = 99%].

Nine studies provided data on the distribution of activation mapping (Figure 3). The predominant targeted activation site in BrS and ERS was the RVOT muscle origin [proportion 0.60; 95% CI (0.30–0.90); *I* ^2^ = 0% and proportion 0.60; 95% CI (0.00–0.31); *I* ^2^ = 30%, respectively]. In LQTS, the dominant targeted activation site was the LV Purkinje origin [proportion 0.75; 95% CI (0.33–1.00)]. In IVF, the targeted activation sites were almost evenly distributed between the RV and LV Purkinje origins [proportion 0.49; 95% CI (0.38–0.60); *I* ^2^ = 0% and proportion 0.46; 95% CI (0.35–0.57); *I* ^2^ = 0%, respectively].

**FIGURE 2:**
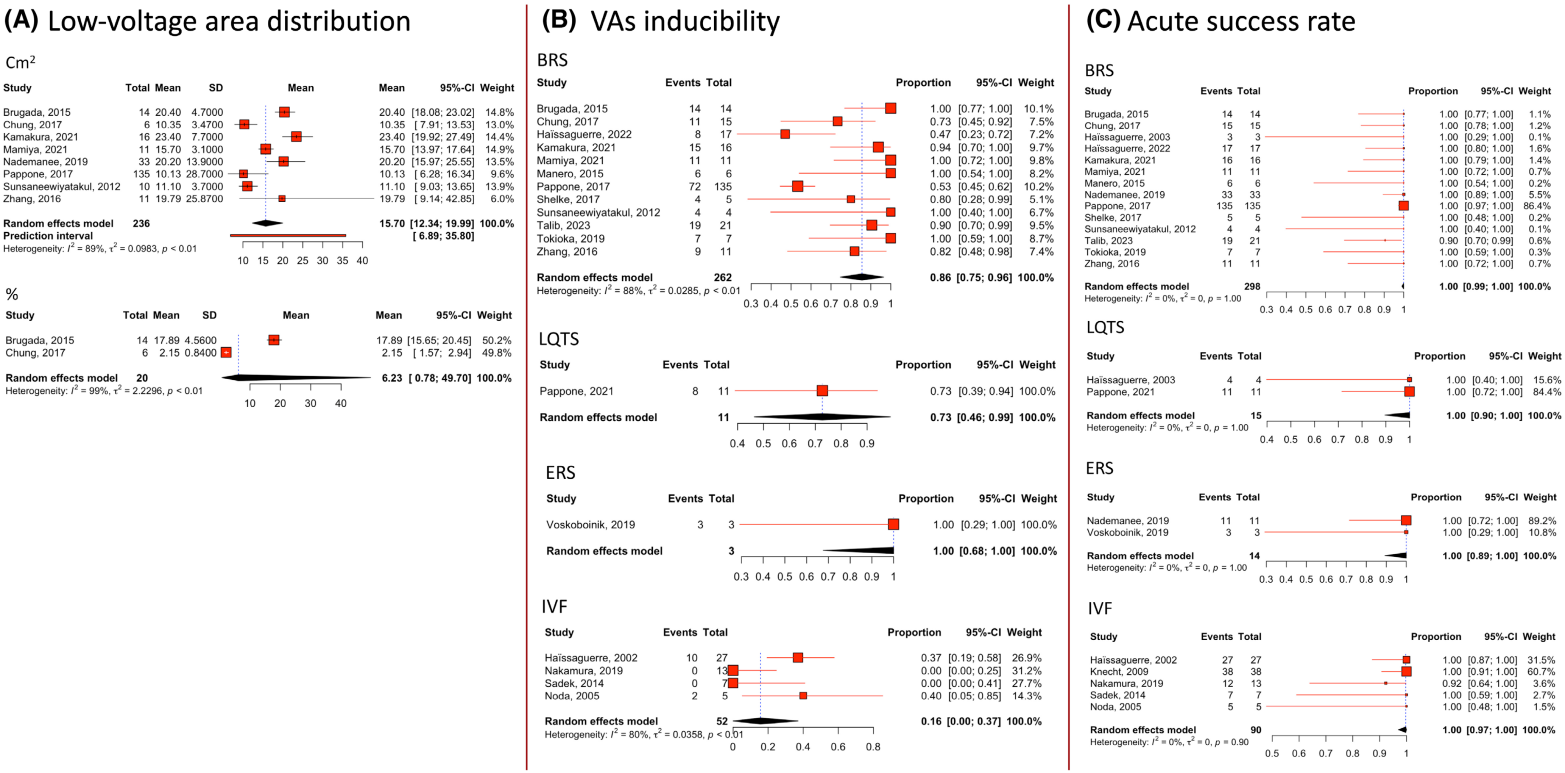
Forest plots prevalence (%) and mean with random‐effect models of the procedural outcomes. (A) Low‐voltage area distribution, (B) VAs inducibility during EPS, and (C) Acute procedural success rate. BrS, Brugada syndrome; CI, confidence interval; ERS, early repolarization syndrome; IVF, idiopathic ventricular fibrillation; LQTS, long‐QT syndrome; VA, ventricular arrhythmia.

Regarding programmed ventricular stimulation (PVS) outcome during electrophysiological study (EPS) (Figure 2B), high VAs inducibility was observed in BrS [proportion 0.86; 95% CI (0.75–0.96); *I* ^2^ = 88%], LQTS [proportion 0.73; 95% CI (0.46–0.99)], and ERS [proportion 1.00; 95% CI (0.68–1.00)], while IVF exhibited a lower inducibility rate of only 16% [proportion 0.16; 95% CI (0.00–0.37); *I* ^2^ = 80%]. The success rate of ablation for all types of IPASs was 100% (Figure 2C).

**FIGURE 3:**
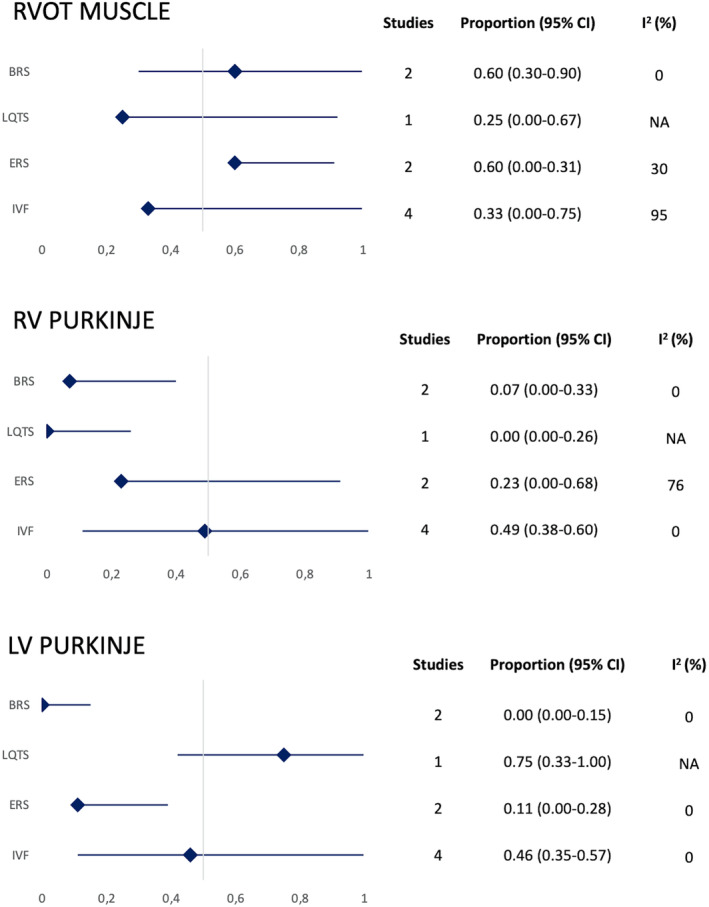
Forest plots prevalence (%) of earliest activation sites distribution. BrS, Brugada syndrome; CI, confidence interval; IVF, idiopathic ventricular fibrillation; LQTS, Long‐QT syndrome; LV, left ventricle; RVOT, right ventricular outflow tract; RV, right ventricle.

**FIGURE 4:**
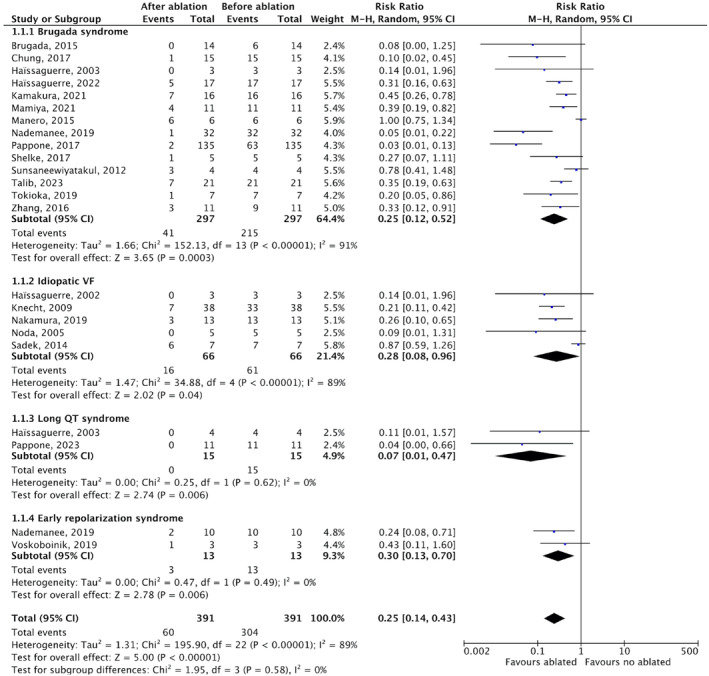
Forest plots risk ratio (RR) with random‐effect models of the outcomes of Ventricular arrhythmia (VA) recurrency after overall ablatio in Inherited primary arrhythmia syndromes (IPASs). CI, confidence interval; M–H, Mantel–Haenszel.

**FIGURE 5:**
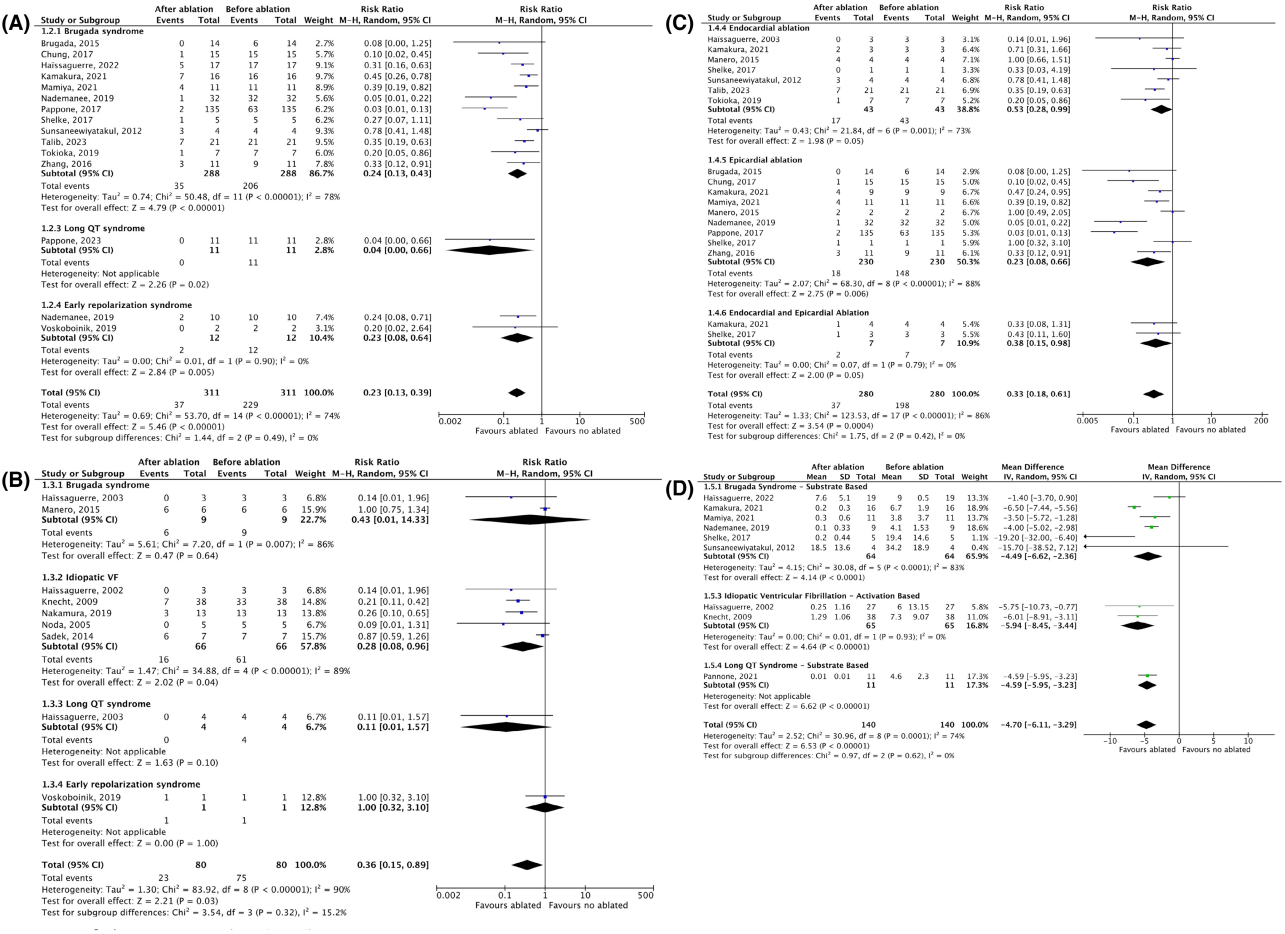
Forest plots risk ratio (RR) and mean difference (MD) with random‐effect models of the outcomes of Ventricular arrhythmia (VA) ablatio in Inherited primary arrhythmia syndromes (IPASs). (A) VA recurrency based on substrate‐based ablation in IPASs. (B) VA recurrency based on activation‐based ablation in IPASs. (C) VA recurrency based on endo‐ and/or Epicardial ablation in Brugada syndrome (BRS). (D) VA burdens. CI, confidence interval; M–H, Mantel–Haenszel.

**FIGURE 6:**
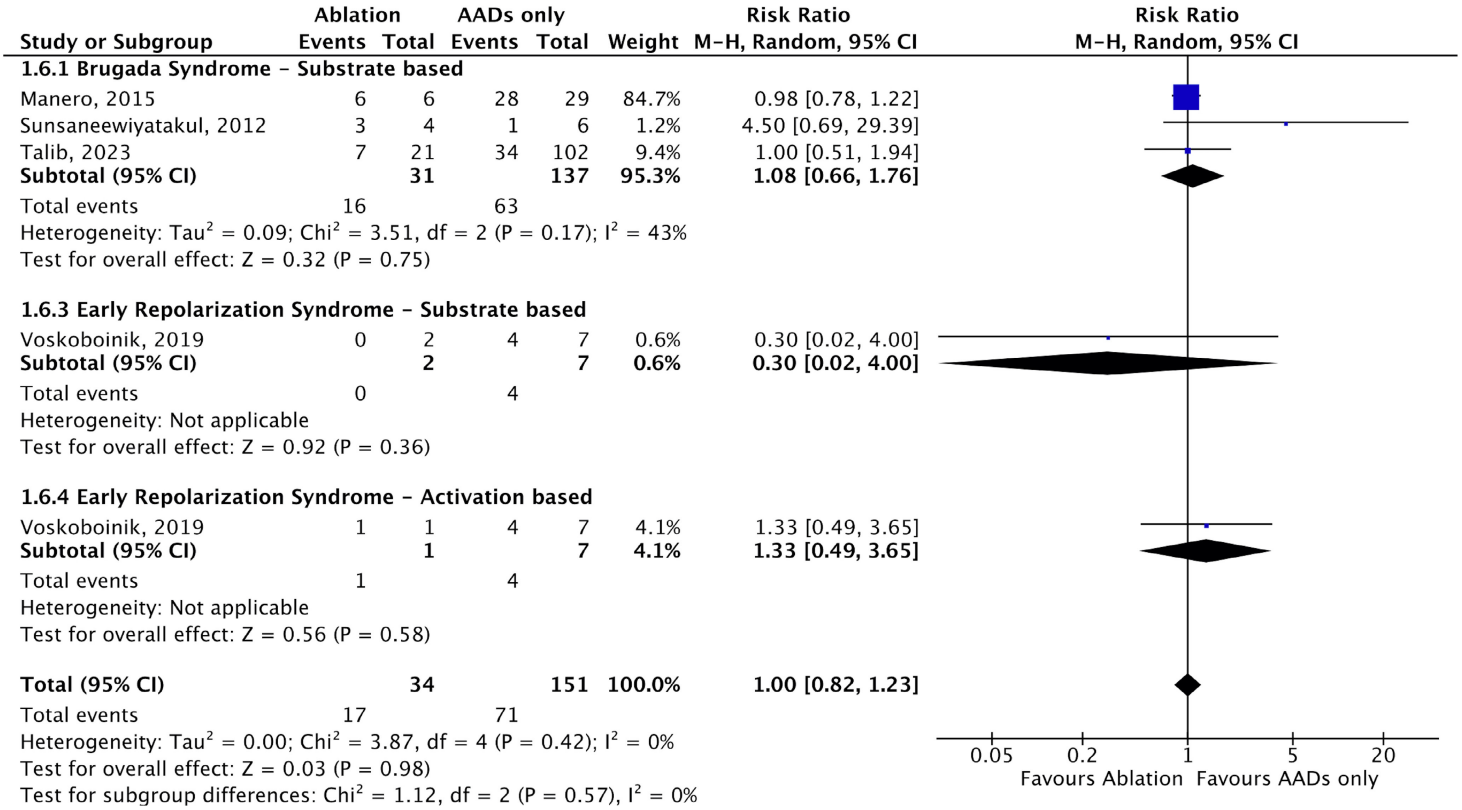
Forest plots risk ratio (RR) with random‐effect models of the outcomes of Ventricular arrhythmia (VA) ablatio compared with anti‐arrhythmic drugs (AADs) only in Inherited primary arrhythmia syndromes (IPASs). CI, confidence interval; M–H, Mantel–Haenszel.

### 3.6 Ablation outcomes

After a mean follow‐up time of 33.5 months, VA ablation demonstrated a significant reduction in recurrent VA events in the entire population of individuals with IPASs and in each subgroup based on the specific type of IPASs [RR 0.25; 95% CI (0.14–0.43); *p* < .001; *I* ^2^ = 89%, Figure 4]. Post‐ablation, all patients discontinued AADs unless they experienced VA recurrence, except LQTS patients, continued beta‐blocker treatment. The same results were observed when the analysis was performed separately for each type of IPASs using substrate‐based ablation [RR 0.23; 95% CI (0.13–0.39); *p* < .001; *I* ^2^ = 74%, Figure 5A]. However, in studies utilizing activation‐mapping guided ablation (Figure 5B), there was no significant difference in VA events before and after ablation in the BrS, LQTS, and ERS subgroups. On activation‐mapping guided ablation, a significant reduction in recurrent VA events was only achieved in the IVF subgroup [RR 0.28; 95% CI (0.08–0.96); *p* = .04; *I* ^2^ = 89%].

Analysis of ablation outcomes based on location in the BrS population revealed that endocardial, epicardial, or combined epicardial and endocardial ablation approaches were equally effective in reducing VA recurrence [RR 0.53; 95% CI (0.28–0.99); *p* = .05; *I* ^2^ = 73%; RR 0.23; 95% CI (0.08–0.66); *p* = .006; *I* ^2^ = 88%; RR 0.38; 95% CI (0.15–0.98); *p* = .05; *I* ^2^ = 0%, respectively, Figure 5C]. The annual burden of VA episodes requiring appropriate ICD therapy also significantly decreased compared to pre‐ablation values [MD –4.70; 95% CI (−6.11–(−3.29); *p* < .001; *I* ^2^ = 74%; Figure 5D]. When compared to a long‐term strategy of AADs, VA ablation without AADs demonstrated comparable outcomes in reducing VA recurrence (*p* =  0.98, Figure 6).

## 4 DISCUSSION

The primary findings of this comprehensive meta‐analysis, representing the first global assessment of its kind, investigating the long‐term outcomes of catheter ablation in IPASs are as follows: (i) CA demonstrates efficacy in reducing VA recurrences across various IPASs, namely BrS, LQTS, ERS, and IVF. (ii) Substrate‐based ablation, with or without activation‐mapping guidance, exhibits favorable therapeutic outcomes in the management of these IPASs. (iii) Activation‐mapping guidance as a standalone approach may be effective solely in IVF cases but not in other IPASs due to a notable recurrence rate disparity. (iv) With the exception of BrS, which predominantly manifests an increased proportion of substrate in the RVOT, each IPAS exhibits an abnormal substrate, likely contributing to the etiology of ventricular arrhythmias. Consequently, routine identification of abnormal substrates within IPASs holds potential clinical value in guiding patient selection for ablation therapy. This approach may be considered even as primary prevention alongside the concurrent implementation of ICD placement. (v) VA ablation significantly reduces annual VA burdens independent of the potential for recurrence.

The previous 2022 ESC Guidelines for the management of patients with ventricular arrhythmias and the prevention of SCD have highlighted the existing gap in evidence regarding the role of endo‐epicardial substrate and/or trigger mapping in identifying locations that may trigger VAs and the subsequent outcome of catheter ablation in primary electrical diseases.^2^ While ICD implantation serves as a preventive measure against SCD, it does not eliminate or reduce the burden of VAs. Consequently, patients may continue to experience psychological, physical, and mental well‐being issues even after receiving ICD shocks.^4^, ^27^, ^28^, ^29^, ^30^, ^31^ This study demonstrates that the inducibility of VAs in IPASs remains relatively high. VA ablation aimed at eliminating abnormal substrates and targeting the earliest activation site, has shown improved acute procedural success rates.

The identification and elimination of abnormal substrates in IPASs were initially reported in the study conducted by Haïssaguerre et al.^11^ This study observed the presence of abnormal substrates in the RVOT and demonstrated promising outcomes following their ablation in patients with BrS. The exact reasons for the presence of arrhythmogenic substrates in IPASs, both endocardial and epicardial, remain unclear. However, studies have suggested that abnormalities in sodium channels, such as those associated with SCN5A gene mutations, may contribute to structural alterations characterized by fibrosis or apoptosis.^32^, ^33^, ^34^, ^35^, ^36^ In the present meta‐analysis, it was found that SCN5A mutations were confirmed in nearly one‐fourth of the IPASs population who underwent genetic testing.

### 4.1 Brugada syndromes

The mechanism underlying VAs in patients with BrS remains a subject of ongoing research and debate. Most studies have consistently identified the presence of a structural arrhythmogenic substrate in the RVOT, particularly in the epicardial region. Two main hypotheses have been proposed to explain the pathophysiological mechanisms involved in BrS: the repolarization theory and the depolarization theory. In their previous study, Nademanee et al.,^37^ reported a local fractioned electrogram (EGM) in the anterior RVOT epicardium, showing that delayed depolarization in the RVOT area contributes to the Brugada ECG pattern. However, a study by Szel and Antzelevitch,^38^ demonstrated that the heterogeneity in phase 2 of the action potential's appearance caused the observed fractionated EGM activity. They proposed that abnormal repolarization was the primary cause of late potentials and fractionated EGMs recorded from the epicardium. Recent research, however, has linked BrS to interstitial fibrosis, the epicardial surface, and decreased gap junction expression in the RVOT.^39^ This aligns with the findings of the meta‐analysis, which showed that both endocardial and epicardial ablation were equally effective in reducing VA recurrence, indicating the presence of arrhythmogenic substrates at these sites.

The findings of the meta‐analysis suggest that activation mapping alone may not be sufficient to accurately assess and guide ablation in patients with arrhythmogenic substrates associated with BrS and other primary electrical diseases. The use of 3D‐EAM, which aids in substrate‐based ablation, appears to provide better precision in identifying and targeting arrhythmogenic substrates. The study revealed that activation mapping of the RVOT identified the earliest activation sites in only about 60% of cases, whereas 3D electro‐anatomical mapping detected arrhythmogenic substrates in the epicardial RVOT in up to 99% of cases. This disparity can be attributed to the infrequent occurrence of spontaneous PVCs that are often challenging to map accurately, especially when attempting to confirm the origin of VF‐triggering PVCs.^35^ The abnormal EGM signals observed in BrS, characterized by low frequency and long duration, differ from the discrete, isolated late potentials commonly observed in fibrotic scars resulting from myocardial infarction.^20^ Circuit elimination through ablation is highly effective in reducing recurrences of scar‐related VT by interrupting abnormal electrical pathways in complex scars. Whereas in BrS, low‐voltage regions reflect localized abnormal conduction rather than complex scars. Ablation targeting these areas may not completely prevent VT recurrences due to underlying pathophysiological abnormalities in BrS.

### 4.2 Early repolarization syndromes

The main difference between BrS and ERS lies in the location of the arrhythmogenic substrate within the heart.^39^ In ERS, the inferior to lateral epicardial walls is considered the most common sites due to higher intrinsic I_to_ density.^40^ This meta‐analysis confirms that the inferior epicardial RV and LV are the regions where ERS arrhythmias are most likely to occur, with a significant distribution of arrhythmogenic substrate. Substrate‐based ablation has shown effectiveness in reducing recurrent ventricular arrhythmias in ERS similar to BrS. Voskoboinik et al.,^25^ reported that 3 out of 10 ERS patients who underwent EPS all proved to have an arrhythmogenic substrate and underwent CA. Among these patients, one experienced recurrence of VAs after ablation guided by activation mapping alone. The remaining seven patients opted not to undergo EPS and thus did not receive ablation. Among them, four experienced recurrent VAs despite receiving AAD therapy.

3D‐EAM has revealed that in patients with ERS, VF rotors, and focal activities are consistently observed at the septum, suggesting their role as drivers and initiators of VF originating from the Purkinje network.^18^ This finding helps explain the involvement of the Purkinje network as a trigger for VF. When the RV serves as the source of drivers, the propagation of electrical current from the thinner RV may not sufficiently excite the septum and LV, leading to sink‐source mismatch and block.^41^ As a result, reentrant rotors tend to anchor at the septum on the epicardium. This highlights the importance of combining substrate‐ and activation mapping in future ablation procedures for ERS, in order to accurately identify the targets for ablation.

### 4.3 Long‐QT syndromes

LQTS is considered an IPAS associated with various ion channel mutations,^42^ which can increase the risk of early afterdepolarizations triggering VAs.^43^ Haïssaguerre et al. demonstrated the effectiveness of eliminating focal triggers through activation‐mapping guided endocardial catheter ablation, resulting in the absence of VAs recurrence in all LQTS patients.^12^ However, this study was limited by a relatively small sample size. Another study by Pappone et al.^5^ reported that only one patient successfully underwent endocardial PVC ablation during follow‐up after epicardial ablation in the entire population, as frequent PVCs are rarely observed in LQTS patients. Hence, the combination of substrate‐ and activation mapping is highly emphasized for the management of LQTS.

Similar to BrS, the arrhythmogenic substrate in LQTS is predominantly found in the epicardial RVOT and other regions of the RV. There have been no reports of arrhythmogenic substrates identified on the endocardium or the LV in LQTS. The abnormal substrate localization may correspond to the anatomical distribution of the autonomic nervous system (ANS) in the RV, where sympathetic fibers are primarily located in the sub‐epicardium. This imbalance in nerve density and chronically increased adrenergic tone has been reported by Pappone et al.,^5^, ^44^ highlighting the role of the ANS in LQTS pathophysiology. Furthermore, the epicardial region contains multipotent progenitor cells that can be activated and differentiate into fibroblasts or adipocytes, leading to the deposition of fibrous and/or fatty tissue in the subepicardial myocardium.^5^, ^44^ This complex histological architecture contributes to microstructural electrical remodeling, making the subepicardial layers more susceptible to electrical instability and affecting the normal repolarization gradient between the endocardium and epicardium. These findings suggest that these pathological pathways contribute to progressive electroanatomic damage, including the development of microstructural fibrosis (characterized by low‐voltage areas), which in turn may contribute to the occurrence of life‐threatening arrhythmias.^16^ Despite the heterogeneity of activation‐mapping findings, the involvement of the Purkinje network in LQTS is believed to contribute to automaticity, reentry, or triggered activity. The exact mechanisms and clinical factors influencing the Purkinje network's role in LQTS remain unclear.^12^ However, current evidence suggests that the epicardial layer plays a significant role in the generation of ventricular arrhythmias in LQTS, emphasizing the importance of substrate‐based ablation. Nevertheless, both studies recommend targeting the sites of triggered ventricular arrhythmias before addressing the arrhythmogenic substrate. This approach is crucial because once triggered, a myocardial substrate with spatially irregular conduction properties can sustain malignant arrhythmias.

### 4.4 Idiopathic ventricular fibrillation

In IVF patients, where no arrhythmogenic substrate was found on the epicardium or endocardium, activation mapping alone was used to guide the ablation procedure. Activation sites were predominantly identified in the Purkinje network, with a small portion originating from the RVOT muscle. In a study conducted by Sadek et al.,^20^ only five patients with Purkinje potentials and seven patients with PVCs originating from the Moderator band were identified. The Purkinje network includes a single right branch that extends into a small portion of the right ventricle, as well as two large left branches with complex branching patterns that supply a larger area of the left ventricle.^45^ There is increasing evidence suggesting that the Purkinje network plays a significant role in initiating and sustaining VF. The precise mechanism is not fully understood, but it is believed that early afterdepolarizations, which are caused by inward calcium currents, are more likely to occur in the Purkinje tissue compared to other regions of the ventricular myocardium. These early afterdepolarizations can contribute to the occurrence of PVCs and the induction of VAs in structurally normal hearts.^15^ This mechanism is further influenced by mechanical strain, leading to membrane depolarization and prolongation of the action potential.^46^, ^47^ Moderator bands are prone to these forces, especially during bradycardia and increased ventricular filling.^20^ Moreover, the Moderator band is rich in autonomic innervation, which may contribute to arrhythmogenic mechanisms, as previously discussed in LQTS.

The findings regarding the mechanisms of VAs in IVF suggest that activation‐mapping‐guided ablation is effective in reducing VAs recurrence, which distinguishes it from other IPASs. This highlights the importance of identifying the specific VAs responsible for the arrhythmias during IPAS ablation.

### 4.5 Clinical impact

It is acknowledged that the currently recommended AADs for IPASs are not widely available in most centers worldwide.^48^ Conversely, widely available CA has uncertain efficacy and is considered a last resort when other therapies have failed. The results of our study highlight the importance of identifying the specific culprit of VAs responsible for IPASs during the ablation procedure. This aspect is fundamental as it has been demonstrated that the majority of IPASs are associated with micro‐electrical remodeling, which can serve as an arrhythmogenic substrate capable of triggering VAs. Therefore, we recommend the utilization of a comprehensive approach in IPAS ablation, combining activation mapping and substrate mapping techniques. It is important to note that while CA may not completely eliminate the potential for recurrent VAs, it has shown significant success in reducing the burden of ventricular arrhythmias. This reduction in VA burden can be of great benefit in mitigating the limitations associated with ICD therapy.

### 4.6 Limitation

This study is subject to the inherent limitations of a systematic review and meta‐analysis. All included studies were observational studies and case series. While these studies were generally of moderate‐to‐high quality, they do not substitute for large‐scale RCTs. Nonetheless, our findings demonstrate consistent results across different cohorts, strengthening the reliability of our conclusions. It is important to acknowledge that non‐randomized studies may be influenced by the learning curve effect, and some studies had small sample sizes, particularly when subgroup analyses were conducted. However, these limitations have minimal impact on the main outcomes, as the effectiveness of the interventions remained consistently demonstrated. Additionally, there is a scarcity of studies directly comparing the outcomes of CA with AADs alone, resulting in relatively low statistical power for these particular comparisons. Nevertheless, based on the available evidence, CA has shown comparable effects to AADs and may offer advantages in mitigating the limitations associated with long‐term AAD therapy.

## 5 CONCLUSION

IPASs have been proven to possess arrhythmogenic substrates and/or arrhythmogenic activations characteristic of their respective types, which can be effectively detected through a synergistic combination of substrate‐based and activation‐based mapping techniques. Moreover, ablation performed at these specific sites has been conclusively shown to diminish VA recurrence and VA burdens, thereby holding promising therapeutic advantages when implemented proactively.

## CONFLICT OF INTEREST STATEMENT

The authors declare no conflict of interest in preparing this article.

## DECLARATIONS

*Ethical approval statement*: No human participant was involved in this study. *Informed consent*: N/A. *Clinical Trial Registration*: N/A. Registry and Registration Number: The study protocol was registered in the prospective international register of systematic reviews under the protocol number CRD42023414057 further reinforcing the systematic and standardized approach employed in this investigation. *Animal Studies*: N/A.

## Supporting information


Data S1.
