# Supplementary material for: Future direction of substrate‐based catheter ablation in Brugada syndrome and other inherited primary arrhythmia syndromes: Systematic review and meta‐analysis
Source: J Arrhythm. 2023 Nov 1;39(6):909–27. doi: 10.1002/joa3.12947 (PMC10692854; doi:10.1002/joa3.12947)

**Supplementary Material**

| **Study** | **Selection** | | | | **Comparability** | | **Outcome** | | | **Total** |
| --- | --- | --- | --- | --- | --- | --- | --- | --- | --- | --- |
|  | **1** | **2** | **3** | **4** | **1** | **2** | **1** | **2** | **3** |  |
| Brugada, 2015 | **🟑** | **🟑** | **🟑** | **🟑** | **🟑** | - | **🟑** | - | **🟑** | 7 |
| Chung, 2017 | **🟑** | **🟑** | **🟑** | **🟑** | **🟑** | - | **🟑** | **🟑** | **🟑** | 8 |
| Haïssaguerre, 2002 | **🟑** | **🟑** | **🟑** | **🟑** | **🟑** | - | **🟑** | **🟑** | **🟑** | 8 |
| Haïssaguerre, 2003 | **🟑** | **🟑** | **🟑** | **🟑** | **🟑** | - | **🟑** | - | **🟑** | 7 |
| Haïssaguerre, 2022 | **🟑** | **🟑** | **🟑** | **🟑** | **🟑** | - | **🟑** | **🟑** | **🟑** | 8 |
| Kamakura, 2021 | **🟑** | **🟑** | **🟑** | **🟑** | **🟑** | - | **🟑** | **🟑** | **🟑** | 8 |
| Knecht, 2009 | **🟑** | **🟑** | **🟑** | **🟑** | **🟑** | - | **🟑** | **🟑** | **🟑** | 8 |
| Mamiya, 2021 | **🟑** | **🟑** | **🟑** | **🟑** | **🟑** | - | **🟑** | **🟑** | **🟑** | 8 |
| Manero, 2015 | **🟑** | **🟑** | **🟑** | **🟑** | **🟑** | - | **🟑** | **🟑** | **🟑** | 8 |
| Nademanee, 2019 | **🟑** | **🟑** | **🟑** | **🟑** | **🟑** | - | **🟑** | **🟑** | **🟑** | 8 |
| Nakamura, 2019 | **🟑** | **🟑** | **🟑** | **🟑** | **🟑** | - | **🟑** | **🟑** | **🟑** | 8 |
| Noda, 2005 | **🟑** | **🟑** | **🟑** | **🟑** | **🟑** | - | **🟑** | **🟑** | **🟑** | 8 |
| Pappone, 2017 | **🟑** | **🟑** | **🟑** | **🟑** | **🟑** | - | **🟑** | - | **🟑** | 7 |
| Pappone, 2023 | **🟑** | **🟑** | **🟑** | **🟑** | **🟑** | - | **🟑** | **🟑** | **🟑** | 8 |
| Sadek, 2014 | **🟑** | **🟑** | **🟑** | **🟑** | **🟑** | - | **🟑** | **🟑** | **🟑** | 8 |
| Shelke, 2017 | **🟑** | **🟑** | **🟑** | **🟑** | **🟑** | - | **🟑** | **🟑** | **🟑** | 8 |
| Sunsaneewiyatakul, 2012 | **🟑** | **🟑** | **🟑** | **🟑** | **🟑** | - | **🟑** | **🟑** | **🟑** | 8 |
| Talib, 2023 | **🟑** | **🟑** | **🟑** | **🟑** | **🟑** | - | **🟑** | **🟑** | **🟑** | 8 |
| Tokioka, 2019 | **🟑** | **🟑** | **🟑** | **🟑** | **🟑** | - | **🟑** | **🟑** | **🟑** | 8 |
| Voskoboinik, 2019 | **🟑** | **🟑** | **🟑** | **🟑** | **🟑** | - | **🟑** | **🟑** | **🟑** | 8 |
| Zhang, 2016 | **🟑** | **🟑** | **🟑** | **🟑** | **🟑** | - | **🟑** | **🟑** | **🟑** | 8 |

Supplementary Table 1: Risk of bias assessment of observational studies included in the meta-analysis according to the Newcastle-Ottawa Scale.

Supplementary Figure 1. Forest plots prevalence (%) with random effect models of Arrhythmogenic substrate distribution.


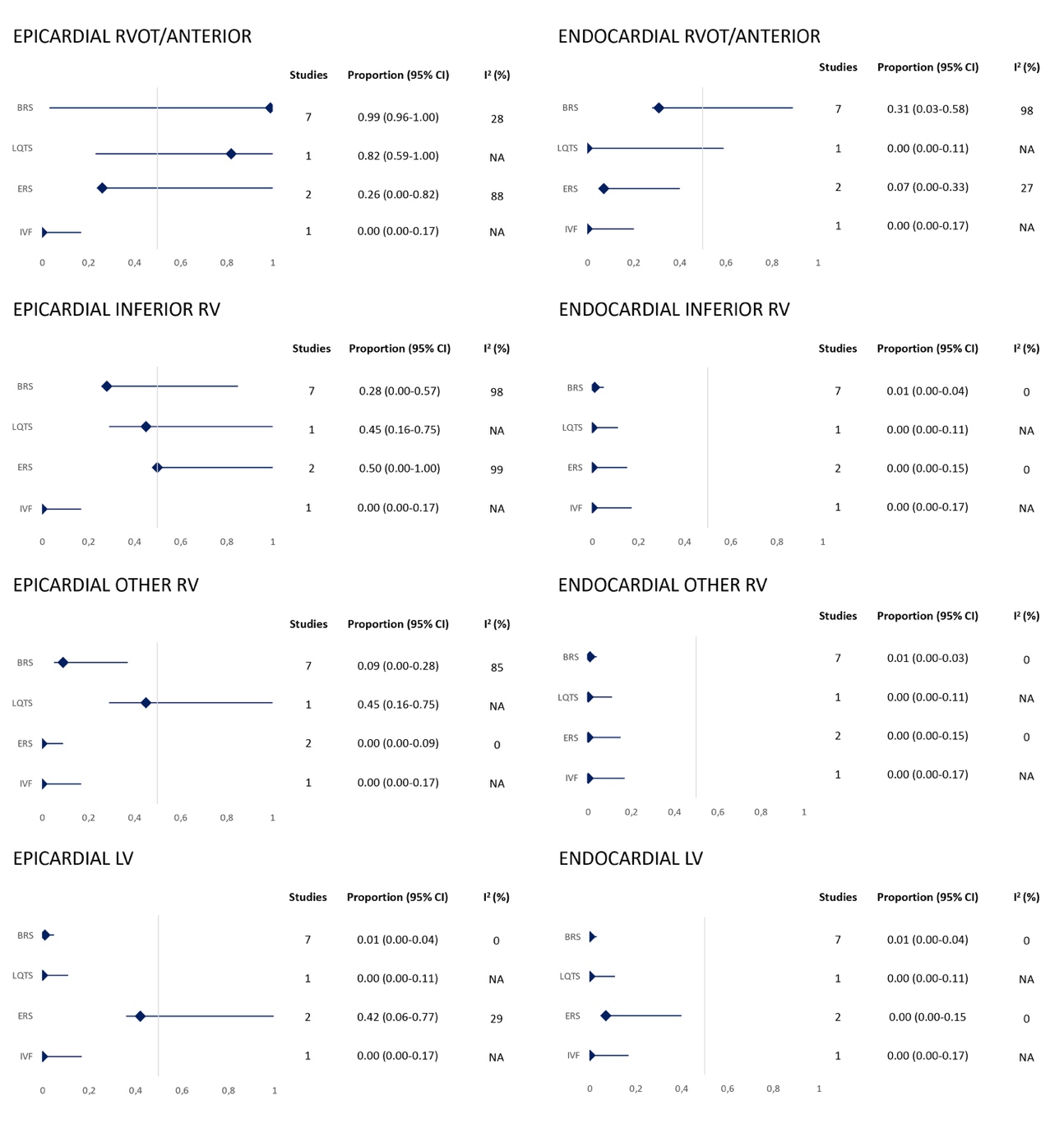
RVOT: right ventricular outflow tract. RV: right ventricle. LV: left ventricle. BRS: Brugada syndrome. LQTS: long-QT syndrome. ERS: early repolarization syndrome. IVF: Idiopathic ventricular fibrillation. CI: confidence interval.

Supplementary Figure 2: (A) Funnel plot of VAs recurrency in overall CA


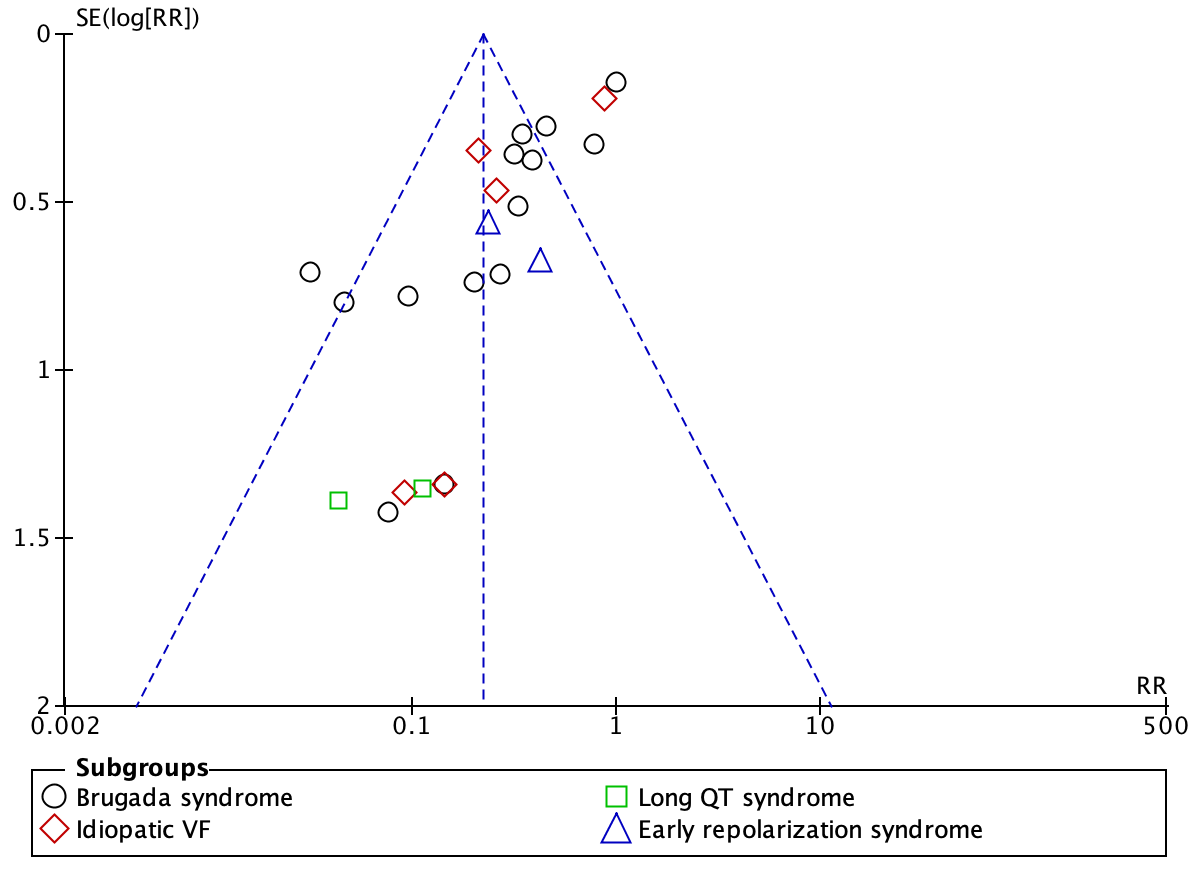


(B) Funnel plot of VAs recurrency based on substrate-based CA


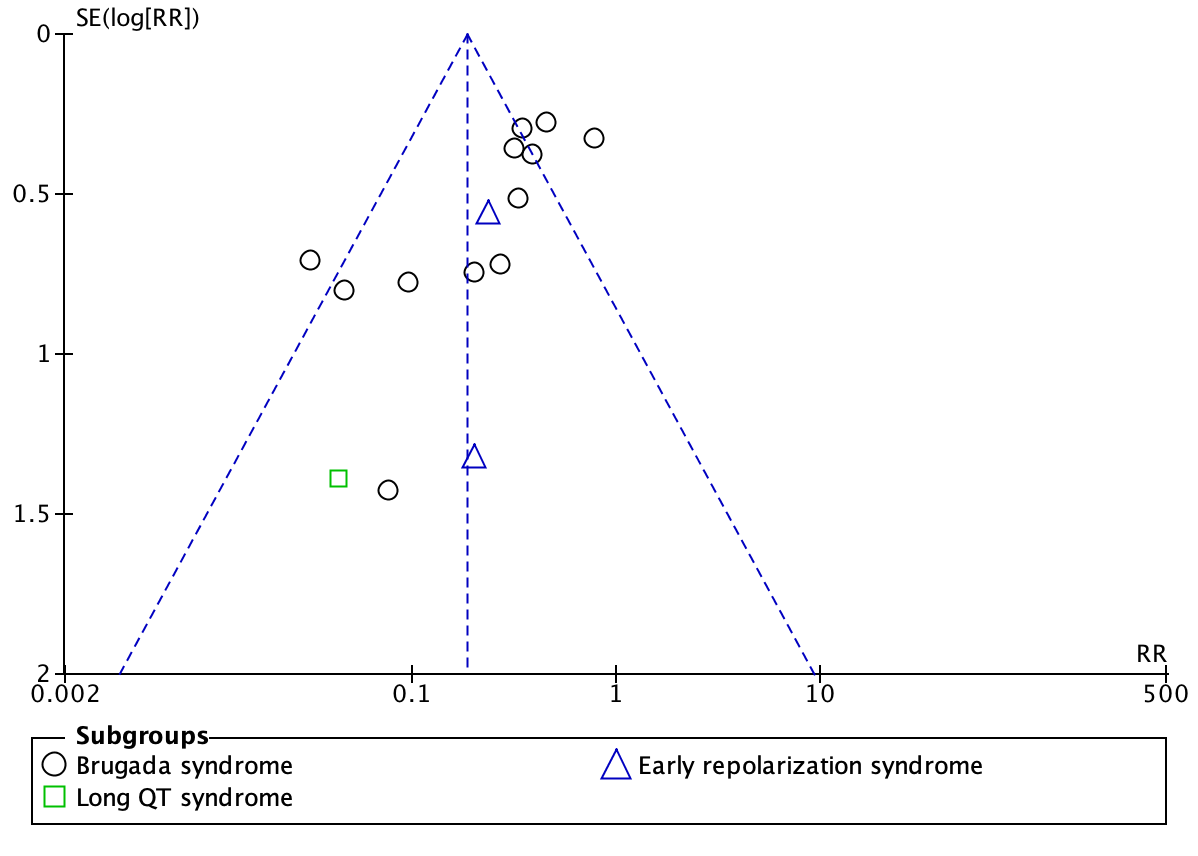


(C) Funnel plot of VAs recurrency based on activation-based CA


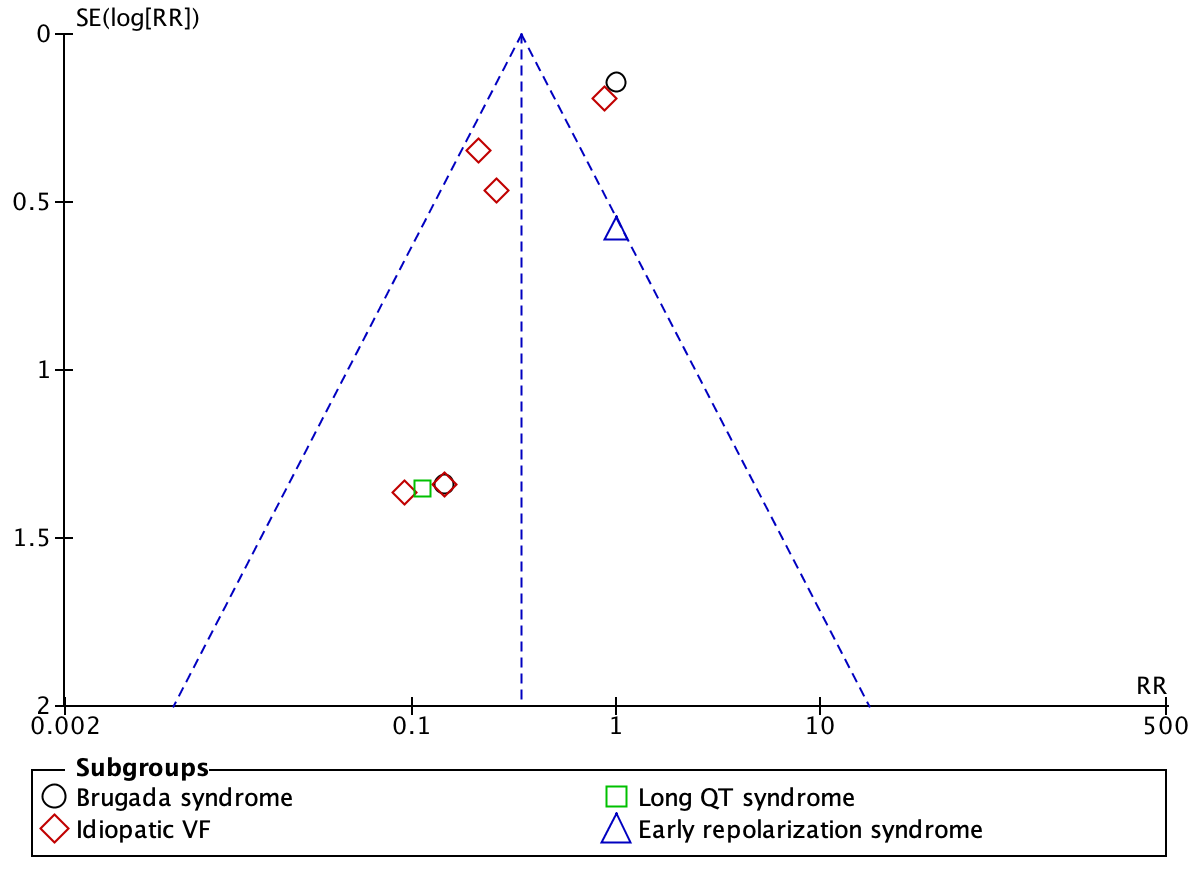


(D) Funnel plot of VAs recurrency based on location of CA (Endocardial and/or Epicardial)


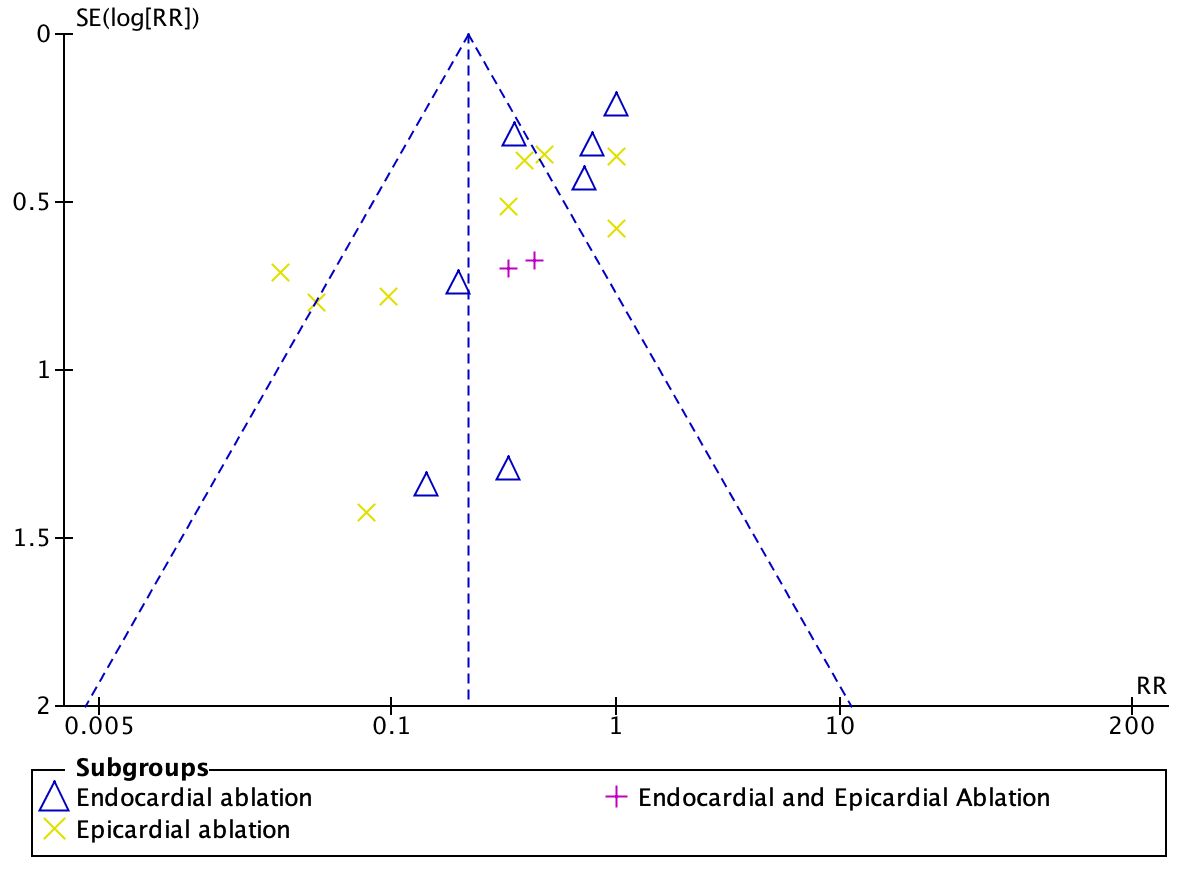


(E) Funnel plot of VAs recurrency CA vs AADs


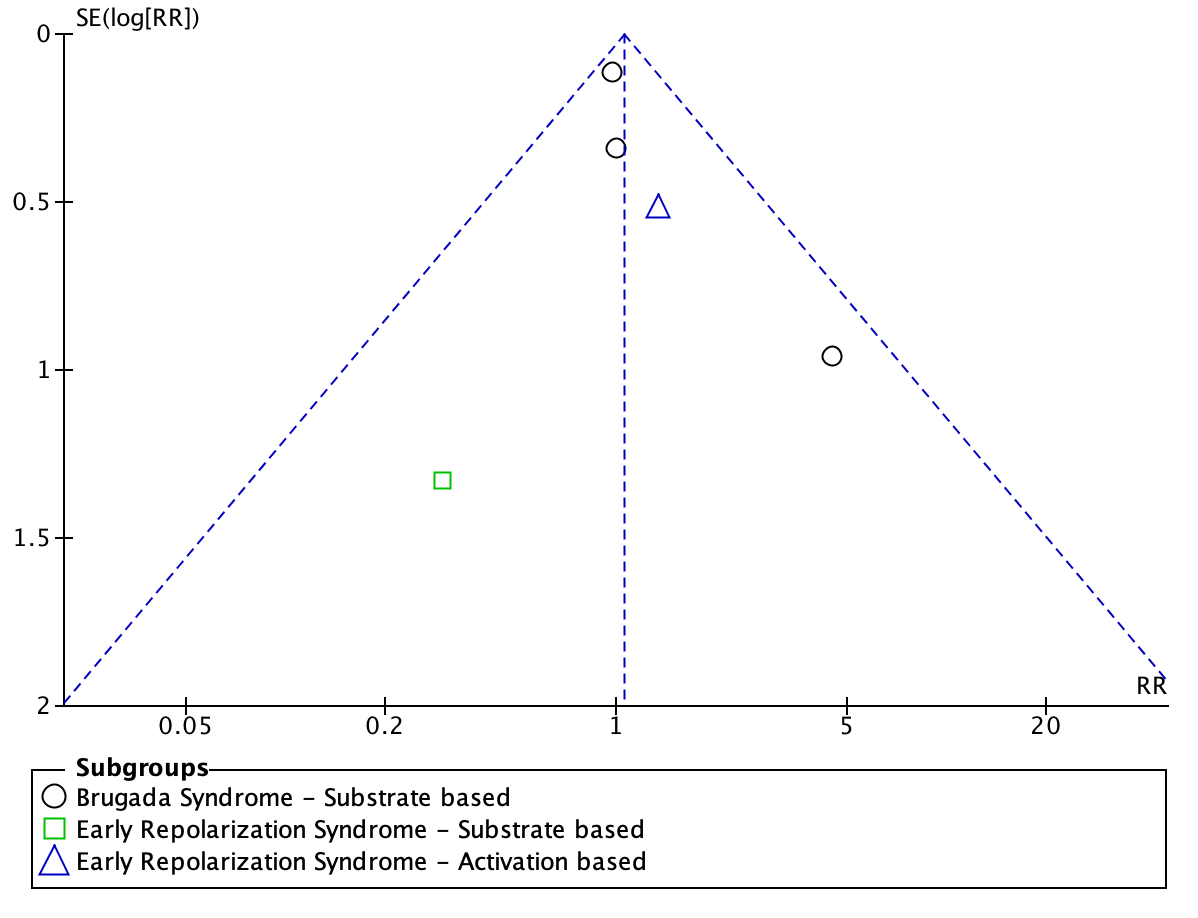


(F) Funnel plot of VA burdens


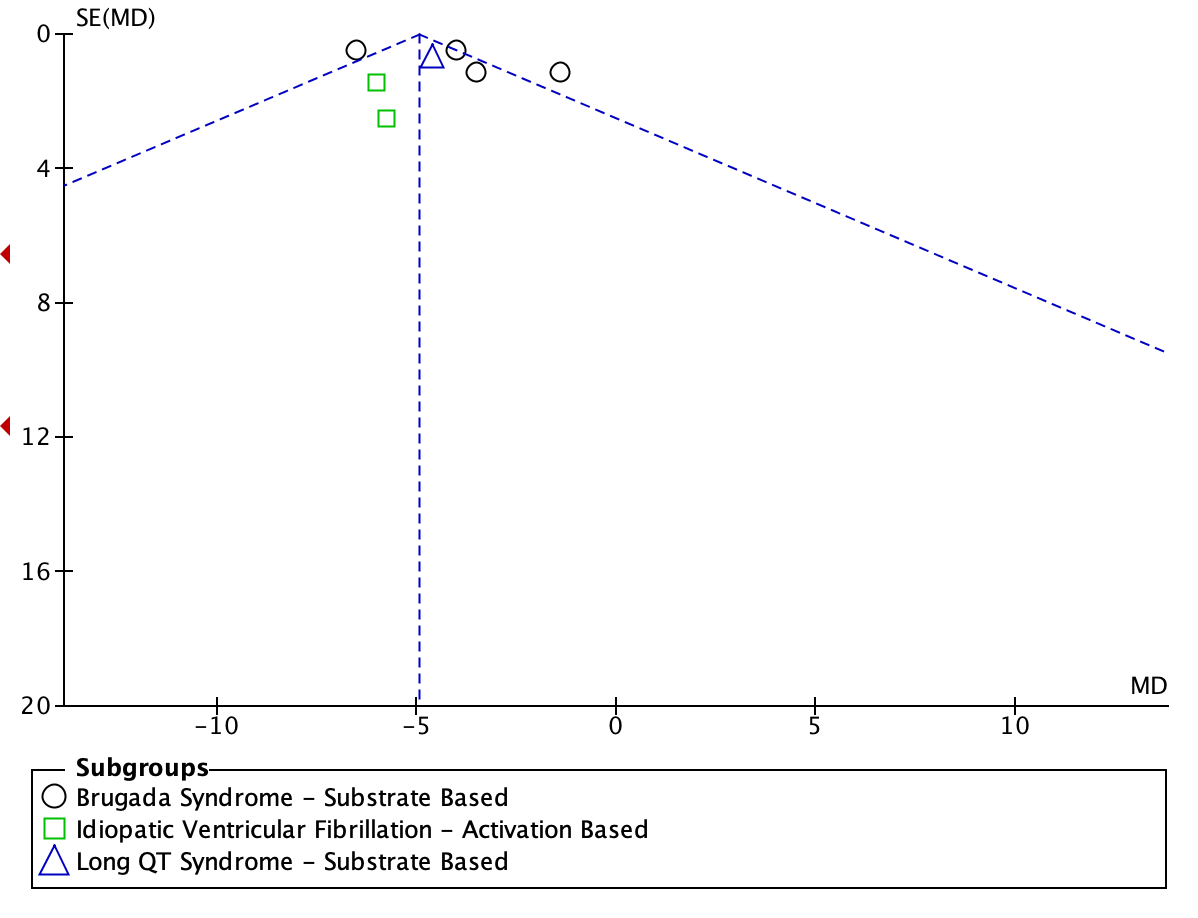


Supplementary Figure 3: (A) Forest and funnel plot of proportion of SCN5A gene mutation in overall IPASs


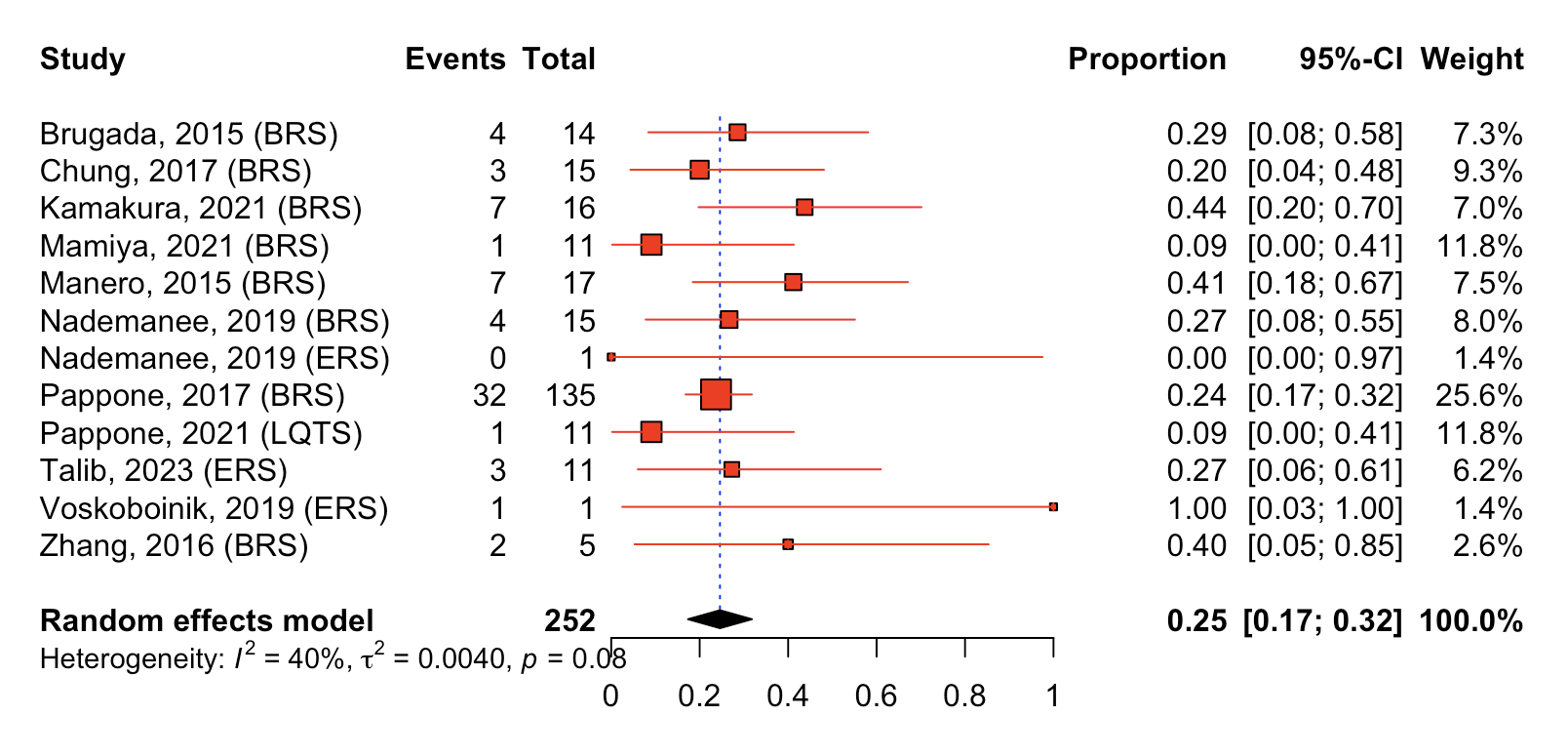


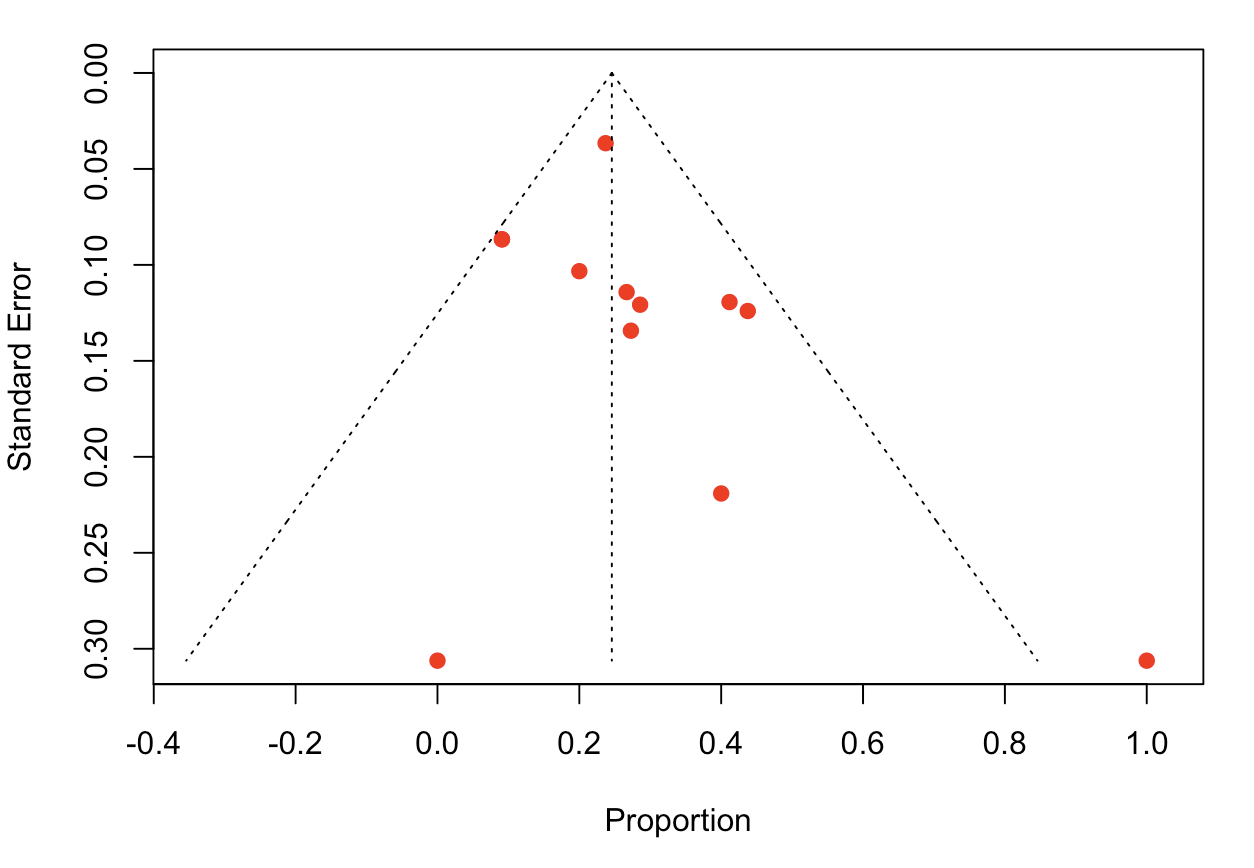


(B) Forest and funnel plot of proportion of SCN5A gene mutation in BrS


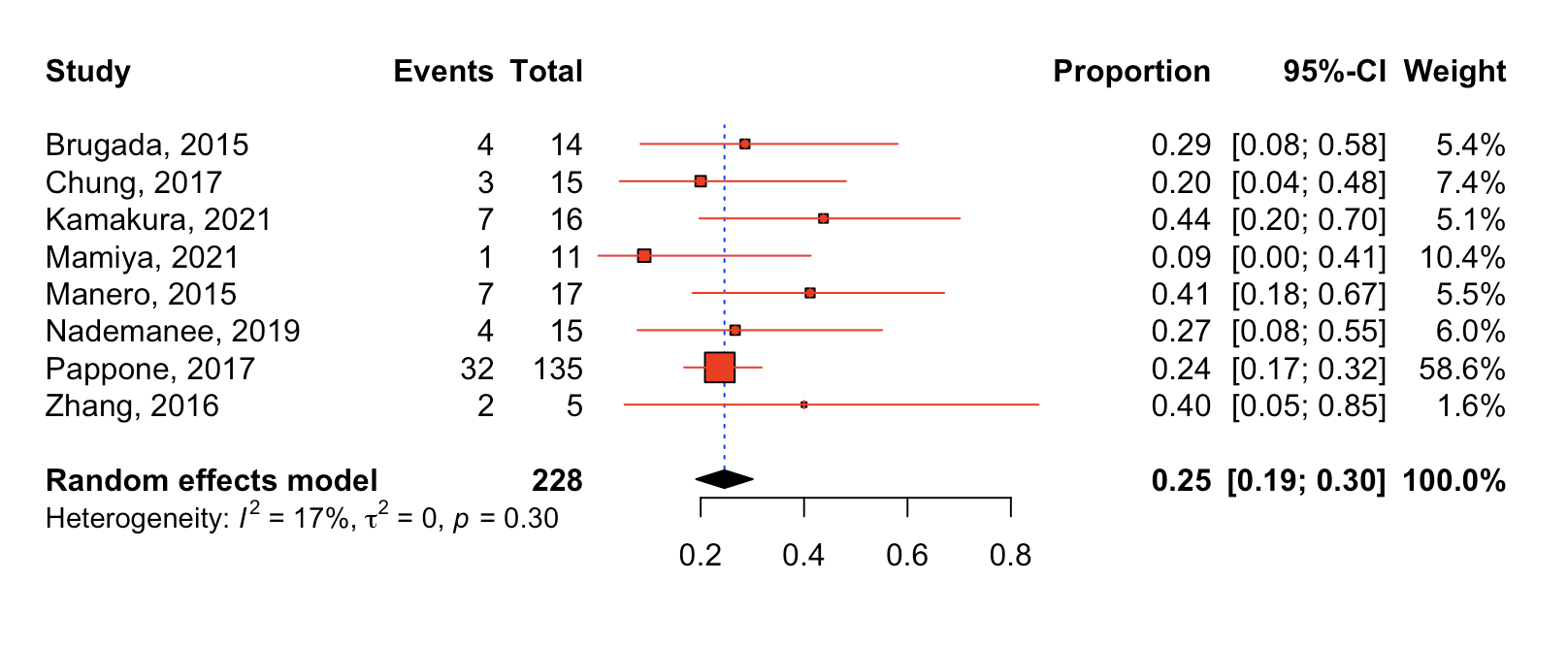

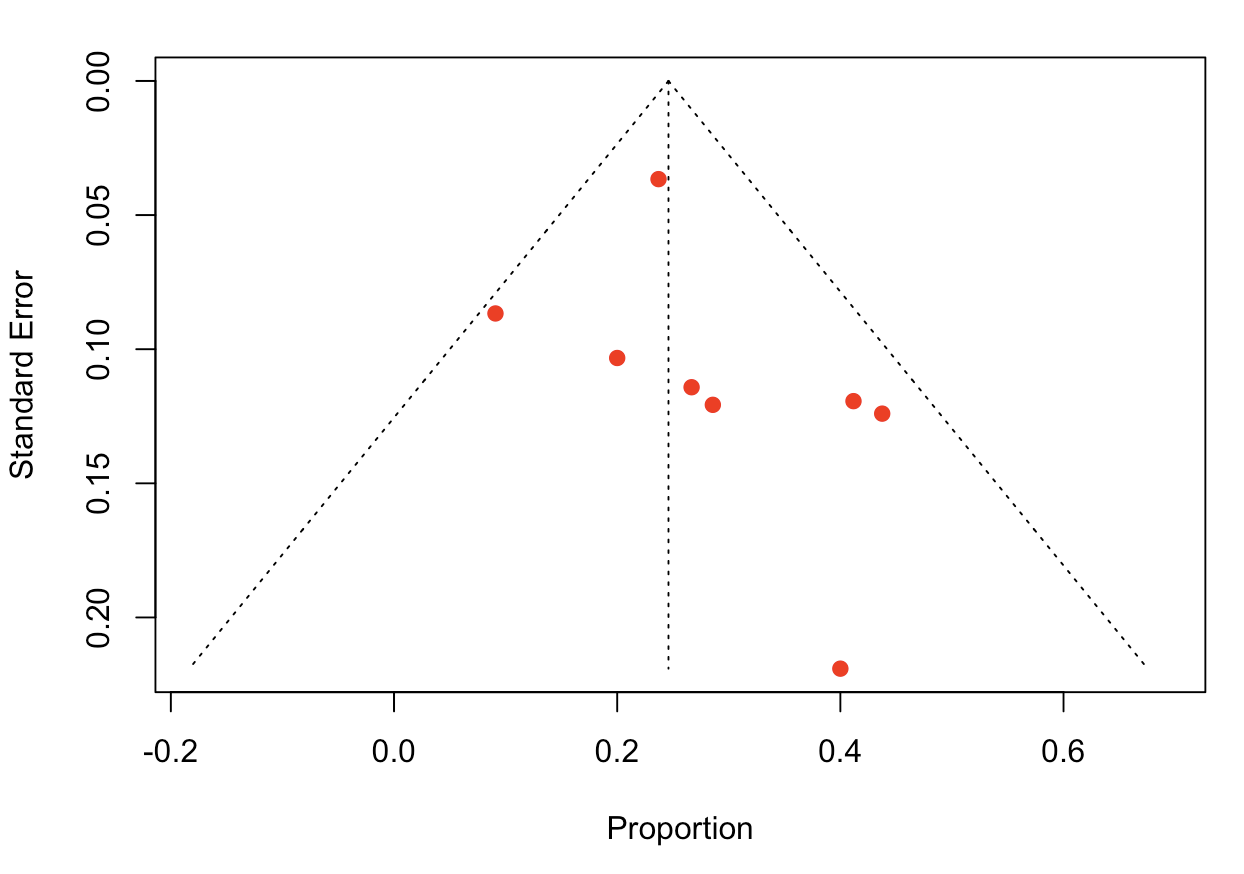

Supplement: Supplementary file 1 — Data S1. [file JOA3-39-909-s001.docx]
